# Population-wide measures due to the COVID-19 pandemic and exposome changes in the general population of Cyprus in March–May 2020

**DOI:** 10.1186/s12889-022-14468-z

**Published:** 2022-12-06

**Authors:** Xanthi D. Andrianou, Corina Konstantinou, Marco A. Rodríguez-Flores, Fragkiskos Papadopoulos, Konstantinos C. Makris

**Affiliations:** 1 Cyprus International Institute for Environmental and Public Health, Cyprus University of Technology, Limassol, Cyprus; 2 Department of Electrical and Computer Engineering, Cyprus University of Technology, Limassol, Cyprus

**Keywords:** Exposome, Pandemic, Modeling, Exposures, Measures

## Abstract

Non-pharmacological interventions (e.g., stay-at-home orders, school closures, physical distancing) implemented during the COVID-19 pandemic are expected to have modified routines and lifestyles, eventually impacting key exposome parameters, including, among others, physical activity, diet and cleaning habits. The objectives were to describe the exposomic profile of the general Cypriot population and compliance to the population-wide measures implemented during March–May 2020 to lower the risk of SARS-CoV-2 transmission, and to simulate the population-wide measures’ effect on social contacts and SARS-CoV-2 spread. A survey was conducted in March–May 2020 capturing different exposome parameters, e.g., individual characteristics, lifestyle/habits, time spent and contacts at home/work/elsewhere. We described the exposome parameters and their correlations. In an exposome-wide association analysis, we used the number of hours spent at home as an indicator of compliance to the measures. We generated synthetic human proximity networks, before and during the measures using the dynamic-${\mathbb{S}}$^1^ model and simulated SARS-CoV-2 transmission (i.e., to identify possible places where higher transmission/number of cases could originate from) on the networks with a dynamic Susceptible-Exposed-Infectious-Recovered model. Overall, 594 respondents were included in the analysis (mean age 45.7 years, > 50% in very good health and communicating daily with friends/family via phone/online). The median number of contacts at home and at work decreased during the measures (from 3 to 2 and from 12 to 0, respectively) and the hours spent at home increased, indicating compliance with the measures. Increased time spent at home during the measures was associated with time spent at work before the measures (β= -0.87, 95% CI [-1.21,-0.53]) as well as with being retired vs employed (β= 2.32, 95% CI [1.70, 2.93]). The temporal network analysis indicated that most cases originated at work, while the synthetic human proximity networks adequately reproduced the observed SARS-CoV-2 spread. Exposome approaches (i.e., holistic characterization of the spatiotemporal variation of multiple exposures) would aid the comprehensive description of population-wide measures’ impact and explore how behaviors and networks may shape SARS-CoV-2 transmission.

**Supplementary Information:**

The online version contains supplementary material available at 10.1186/s12889-022-14468-z.

## Introduction

On March 11 2020, the World Health Organization declared the coronavirus disease (COVID-19) caused by the severe acute respiratory syndrome coronavirus 2 (SARS-CoV-2) a pandemic [1]. By that time, the epidemic that had started in China, with the first cases reported at the end of 2019/beginning of 2020, had spread in different countries with increasing numbers reported in the European Region. The first COVID-19 cases in Cyprus were reported on March 9, 2020 among travelers (imported cases) and as of May 3, 2020 among the 873 cases that had been reported, the majority (82.9% of those with known origin of infection) were locally acquired [2]. During the same period, different non-pharmacological interventions (NPI) were implemented to lower the risk of transmission, including the closure of schools (since March 10, 2020), closure of public spaces (since March 13, 2020) and stay-at-home orders (since March 24, 2020), generally referred to as “lockdown” measures [3]. The mandatory measures that were implemented for specific periods of time were accompanied by more general recommendations and guidelines to stay at home or work from home when possible, physical distancing at working places and in places of social activities and with specific measures e.g. for safety at the workplace [3–6].

The implementation of the physical distancing measures and especially the implementation of stay-at-home orders are anticipated to change people’s routine and lifestyle. These changes include decreases in the number and duration of contacts among individuals and changes in the context social contacts are made [7]. These changes can be described using different methods including human proximity networks, i.e., time-varying graphs that represent the proximity among humans moving in a physical space, which have been used to model contact patterns [8–15].

Besides the changes in lifestyle, social interactions and the decreasing number of contacts, spending less time at the workplace, less time commuting and more time indoors are some of the direct lifestyle changes due to the NPI measures [16]. Changes in lifestyle are directly and indirectly linked to specific environmental exposures and therefore, the compliance with the stay-at-home orders or general recommendations that modified the lifestyle were expected to impact the suite of exposures individuals are subject to. These modifications of populations’ and individuals’ exposomes, i.e. the sum of exposures through one’s lifetime, can be comprehensively characterized using the methodological framework of the human exposome and its tools [17, 18]. Studying changes in the individuals’ exposomic profile and population-level effects as a result of the pandemic NPI response measures would allow for better assessing links between exposures, lifestyle and behaviour changes, and their downstream short- and longer-term health effects [17].

In order to describe the possible changes in the exposomic profile of the general population in Cyprus during the first wave of the pandemic (March–May 2020) when a series of population-wide measures were implemented, the Exposome@home|COVID-19 study was set up. The specific study objectives were:

i. to describe the environment, diet, behaviour and lifestyle choices of the general Cypriot population, during the implementation of the population-wide measures, as part of the pandemic response measures, by adopting the methodological context of the human exposome,
ii. to describe the compliance of the general population of Cyprus to the imposed population-wide pandemic measures, and
iii. to apply state-of-the-art probabilistic network models for infectious disease modelling projections using the exposome dataset of the Cypriot population.

## Methods

### Study design

The Exposome@home|COVID-19 study comprised of an online survey using snowball sampling. The survey questionnaire was administered during the time physical distancing measures and stay-at-home orders were implemented during the 1^st^ wave of the COVID-19 pandemic (30 March-4 May 2020). Respondents were informed about the study through mailing lists and through posts and advertisements on social media and they were also invited to forward information about the study and the questionnaire to their contacts. The study was approved by the Cyprus National Bioethics Committee (Decision number 2020.01.52). The CHERRIES checklist for reporting results of Internet E-Surveys is available in the Supplementary material.

### Questionnaire

The questionnaire included different exposome components to collect information ranging from general respondent characteristics, e.g., demographics and habits/lifestyle, as well as information on the number of contacts and the time spent on different locations before and during the implementation of the measures (more information about the questions can be found in the Supplementary material). Based on the classification by Wild (2005), each exposome component and its specific questionnaire variables are associated with a distinct exposome domain (Table 1).

**Table 1:** Exposome domains, groups of components and variables used in the study questionnaire

Exposome domain	Questionnaire component	Variables	Time frame of measured exposome parameters
**Internal**	Participant characteristics (demographics, anthropometrics)	Age, sex, body mass index (BMI)	Background
	Anxiety and quality of sleep	STAI score, sleep efficacy	During the measures
	Health status	Chronic disease presence, flu vaccination	Background
**Specific external**	Habits	Smoking frequency, second-hand smoking, alcohol consumption frequency	During the measures
	Social activities	Digital communication with friends/family/work Screen time	During the measures
		Hours spent in house Number of contacts at home/work/other, and number of hours working at home/work/other Age of contacts and belonging to a vulnerable population group (i.e., being > 60 years-old, or having a chronic disease or being pregnant)	Before and during the measures
	Physical activity and diet habits	30 min of exercise frequency, consumption frequency of specific food groups	During the measures
	Household cleaning activities	Bathroom cleaning, mopping, kitchen cleaning, other surfaces cleaning	During the measures
	Personal hygiene	Handwashing with soap/antiseptic	Before and during the measures
**General external**	Place of residence and socio-economic status	Area of residence, education level, marital status, occupation	Background

### Statistical analysis

To account for differences between the study population and the Cypriot population distribution by age, sex, and geographical district, as well as to allow for extrapolation of the study estimates to the Cypriot population, we weighted the survey population using the raking method [19]. To calculate the weights, we used the most recent (2019) population age and sex estimates by each geographical district available by the Statistical Service of Cyprus [20] (Table S1).

Data was described using the weighted and raw frequencies and percentages for the categorical variables and means (standard deviations) or medians (interquartile ranges) for the continuous variables. The total number of contacts before and during the measures was defined as the sum of the contacts at home, at work and elsewhere. Individual responses with a number of contacts above the 99^th^ percentile of the number of contacts reported by place (home/work/elsewhere) before and during the lockdown were imputed to the 99^th^ percentile value of the respective distribution of all responses. A summary of the number of responses imputed and the 99^th^ percentile values in each category is available in the supplementary material (Table S2).

Compliance to NPI measures was described with the number of hours spent in different places and the number of contacts before and during the measures. The absolute difference and the percent change in the number of contacts and the number of hours spent at different places between the period before the measures and during the measures were calculated. When the number of hours spent at home, at work, working from home and commuting or the number of contacts before the measures was 0, the percent change was estimated by adding 1 in both the before and during values for computation purposes.

Respondents were asked to provide information for up to eight contacts of their household and note their age and whether these contacts belonged to a vulnerable population group, i.e., being > 60 years-old, or having chronic disease, or being pregnant. We estimated the mean age of these contacts and the total number of vulnerable household contacts per respondent. We described the correlation between the age of each respondent and the mean age of their household contacts and the number of vulnerable contacts using heatmaps.

Weighted Pearson correlation coefficients were calculated for the continuous variables including, e.g., contacts and hours spent in different places before and during the measures, dietary habits and anxiety and sleeping patterns, age and BMI.

We used the number of hours spent at home to construct an indicator of compliance based on the crude increase in number of hours spent at home (any increase). In an exposome-wide association analysis (ExWAS), we used weighted logistic regression models to estimate the odds ratio of staying at home more hours during the measures (i.e., the odds ratio of complying with the measures) and respondents’ exposome characteristics and habits before the measures. In a second set of models, we used the number of hours spent at home as the outcome of linear regression models and explored associations with the exposome characteristics. In both sets of models, we performed univariable and multivariable analysis adjusted for age and sex. In the linear regressions, all continuous covariates were scaled and centered. We considered significant the models for which the false discovery rate-adjusted *p*-value was below or equal to 0.1. We used the Benjamini-Hochberg methods to adjust the *p*-values per group of models (e.g., logistic regression univariable analyses and multivariable analyses, separately).

The statistical analysis of the survey responses was conducted in R (version 4.1) using RStudio (version 1.4.1717) [21, 22]. A list of the packages used in the analysis is available in the Supplementary Material.

### SARS-CoV-2 infection transmission simulations on synthetic human proximity networks

We generated synthetic temporal human proximity networks for each setting (home, work, elsewhere) before and during the measures assuming that all respondents were interacting with each other. We used the dynamic-${\mathbb{S}}$^1^ model [11] to generate the synthetic networks and simulate SARS-CoV-2 transmission (Supplementary Material: The dynamic-𝕊1 model).

In the dynamic-S1 model time is slotted. For each network corresponding to a specific setting, we determined the number of time slots by dividing the average of the respondents’ reported hours in the setting with the time slot duration, which we assumed to be 5 min. Respondents with missing reported contacts were excluded. The model was then tuned so that the average node degree (number of contacts per individual) over all time slots in each setting was approximately the same as the average reported number of contacts in the Exposome@home survey (see the Supplementary Material for details). The generated temporal networks for the work, elsewhere and home settings were then combined to form a single temporal network corresponding to the contact patterns of an average day before or during the measures. The analysis was conducted twice, once using the raw data on the number of contacts reported by the respondents and once after normalization of the number of contacts to the averages per age group reported in the POLYMOD survey to account for outliers and possible overestimation in the number of contacts reported by the Exposome@home|COVID-19 respondents [23]. In the main text, we present results for the normalized data (non-normalized results are in the Supplementary Material).

In order to simulate SARS-CoV-2 transmission before and during the measures, we generated 15 synthetic temporal networks before measures and 59 synthetic temporal networks during measures, which mirror the periods of 15 days between the first reported case and the timing of the measures plus the 59 days during which the measures were enforced in Cyprus (9 March -24 March 2020 and 24 March – 21 May 2020, respectively). Using a dynamic Susceptible-Exposed-Infectious-Recovered (SEIR) process, we simulated the transmission of SARS-CoV-2 on the total 74 generated networks. A brief description of the SEIR process can be found in the Supplementary Material. We ran 50 dynamic SEIR processes on the synthetic networks and estimated the reproduction number (R_t_). In the simulations, we chose a different source infected node (respondent) at random in each run with the following simulation parameters: (i) infection probability σ, as the inverse of the incubation period (3 days selected from the range of 3–6 days that has been reported [24–26]); (ii) the recovery probability γ, set as the inverse of the infection duration (14 days as reported by WHO 2020, [27]); and (iii) the exposed probability β, which we set according to the formula R_0_ = β/γ [14], using R_0_ of 3.26 and its lower and upper interval (2.58 and 4.01, respectively) (COVID-19 surveillance report, Cyprus Ministry of Health, [28]). R_t_ is the expected number of new infections caused by a single infected individual at time t. If R_t_ ≤ 1, the outbreak would die out; if R_t_ ≥ 1, a sustained outbreak is likely [29]. We computed R_t_ in the simulations using the formula ${\mathrm{R}}_{\mathrm{t}}={\mathrm{I}}_{\mathrm{t}}/\left(\sum_{\mathrm{s}=1}^{\mathrm{t}}{\mathrm{I}}_{\mathrm{t}-\mathrm{s}}{\mathrm{w}}_{\mathrm{s}}\right)$, where I_t_ is the number of new infected cases on day t and w_s_ is the probability that s days separate the onset of symptoms in an infected individual and the onset of symptoms in its infector—the serial interval— [30], computed empirically from the simulations (Supplementary Material). The R_t_ equation can be interpreted as the average number of secondary cases that each symptomatic individual at time t would infect, if the conditions remained as they were at time t.

## Results

**Fig. 1:**
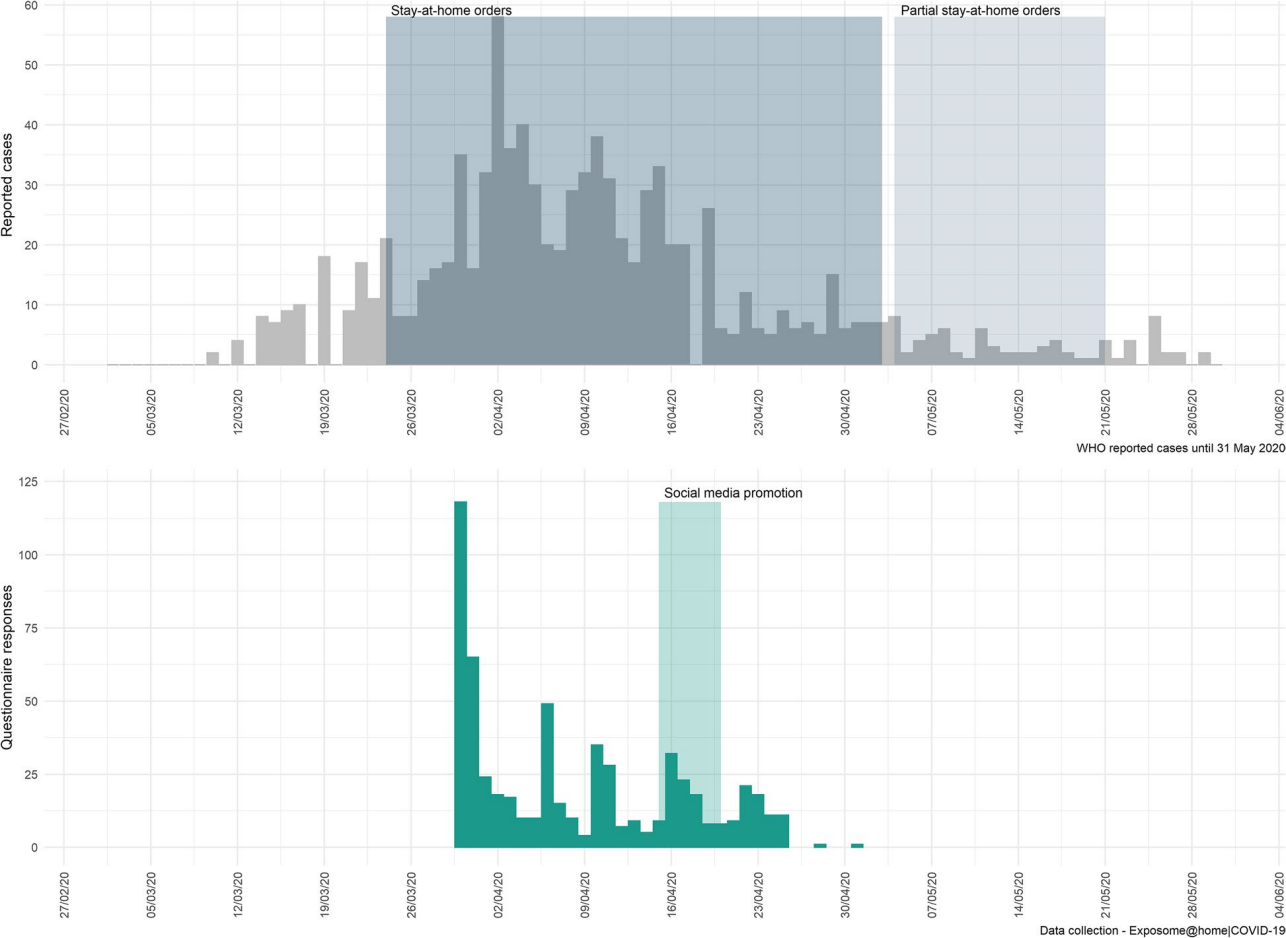
Reported COVID-19 cases in Cyprus until June 1, 2020 and number of questionnaire responses between March 30 and May 1, 2020. The blue box indicates the period during which stay-at-home orders (lockdown) were in place and the light grey box indicates the period of gradual relaxation of the measures (4–21 May, 2020), as reported by the European Center for Disease Control and Prevention [3]

In total, 597 responses were submitted (March 30 – May 01, 2020) (Fig. 1). The highest number of responses was observed in the first day of the dissemination of the questionnaire and then another peak followed when the social media advertisements were placed (Fig. 1).

### Exposomic profile of the study respondents before and during the spring 2020 measures

The parameters of the exposomic profile of the study respondents were categorized in the internal and the external (general and specific) exposome domains (Table 1).

#### Internal exposome domain

Of the 597 respondents of the survey, 594 were included in the weighted analysis after excluding three with unknown sex. Overall, 369 were females and 225 males (62.1% vs 37.9%) with a weighted frequency of 283 females and 311 males (52.4% vs 47.6%). Mean population age was estimated at 45.7 years (standard error (SE) 0.5) and the weighted median BMI was 24.7 kg/m^2^ [95% interquartile range (IQR): 22.3, 27.9] (Table 2, Table S3).

**Table 2:** Weighted estimates of the internal exposome domain characteristics of the Exposome@home|COVID-19 study population, Cyprus, 2020

	**Overall**	**Females**	**Males**
	594	311	283
**Age (mean (SE)) in years**	45.7 (0.5)	40.8 (1.3)	51.1 (1.3)
**Age group (% [95% CI])**			
18–29 years	22.5 [21.9, 23.1]	32.1 [28.7, 35.6]	12 [8.2, 15.8]
30–39 years	20.5 [20, 21.1]	21.8 [17.8, 25.7]	19.2 [15.1, 23.3]
40–49 years	15.9 [15.5, 16.4]	14.9 [11.5, 18.3]	17 [13.4, 20.7]
50–59 years	15.2 [14.8, 15.6]	15.6 [12.2, 19]	14.8 [11.1, 18.6]
60–69 years	13.3 [12.9, 13.6]	10.6 [6.3, 14.9]	16.3 [11.5, 21]
70 + years	12.5 [10.2, 14.8]	5 [-0.7, 10.7]	20.6 [14.7, 26.6]
**BMI (median [IQR]) kg/m**^**2**^	24.7 [22.3, 27.9]	23.7 [20.8, 27.1]	25.6 [23.5, 28.5]
**STAI score category (% [95% CI])**			
High anxiety	64.6 [58.9, 70.2]	72.5 [66.1, 78.9]	55.9 [46.6, 65.1]
Moderate anxiety	17 [12.8, 21.2]	18.2 [12.5, 23.9]	15.7 [9.9, 21.6]
No or low anxiety	18.4 [13.7, 23.2]	9.3 [4.9, 13.8]	28.4 [19.9, 36.9]
**Minutes to sleep score (% [95% CI])**			
0 (< 16 min)	53.2 [48.3, 58.1]	40.6 [34.3, 46.9]	67 [59.4, 74.5]
1 (16–30 min)	11.3 [7.9, 14.6]	13.1 [8.3, 18]	9.3 [4.9, 13.7]
2 (31–60 min)	26.4 [22, 30.8]	36.5 [30.1, 43]	15.2 [9.5, 21]
3 (> 60 min)	9.2 [6, 12.4]	9.8 [6.3, 13.2]	8.5 [3.2, 13.9]
**Sleep duration (% [95% CI])**			
0 (> 7 h)	62.9 [57.3, 68.6]	66.3 [60, 72.5]	59.3 [49.8, 68.7]
1 (6–6.99 h)	23.9 [18.8, 29]	20.6 [14.9, 26.2]	27.6 [18.8, 36.4]
2 (5–5.99 h)	9.4 [6.7, 12.1]	9.4 [5.8, 13.1]	9.4 [5.2, 13.7]
3 (< 5 h)	3.7 [1.5, 6]	3.8 [1.6, 5.9]	3.7 [-0.3, 7.7]
**Habitual sleep efficacy score category (% [95% CI])**			
0 (> = 85)	62.7 [57.4, 68.1]	53.3 [46.3, 60.3]	73.2 [65.6, 80.9]
1 (75–84)	17.9 [14.2, 21.5]	21.7 [16.3, 27.2]	13.5 [8.9, 18.2]
2 (65–74)	10.7 [7.1, 14.3]	13.6 [8, 19.3]	7.5 [3.3, 11.7]
3 (< 65)	8.7 [5.2, 12.3]	11.4 [6.4, 16.4]	5.7 [0.7, 10.8]
**Health status (% [95% CI])**			
Very good	55.5 [49.6, 61.4]	55.3 [48.5, 62]	55.8 [46, 65.5]
Good	37.7 [32, 43.4]	37.9 [31.2, 44.5]	37.5 [27.8, 47.2]
So and so	5.6 [3.5, 7.6]	6.2 [3.4, 9]	4.8 [1.6, 8]
Bad	1.2 [-0.6, 3.1]	0.6 [-0.6, 1.9]	1.9 [-1.8, 5.6]
**Chronic diseases (% [95% CI])**			
1	29.5 [24.5, 34.5]	32.1 [26.2, 37.9]	26.7 [18.4, 34.9]
2 or more	28.6 [23.5, 33.6]	31 [25, 37.1]	25.9 [17.5, 34.2]
None	41.9 [36.5, 47.4]	36.9 [30.6, 43.2]	47.5 [38.1, 56.9]
**Influenza vaccine (%)**			
No	63.8 [58.3, 69.4]	64.4 [58, 70.8]	63.2 [54, 72.5]
Yes	22.4 [17.3, 27.5]	20.3 [14.6, 26]	24.8 [15.8, 33.7]
I don’t know/I don’t remember/I’m not sure	13.7 [11, 16.4]	15.3 [11.2, 19.5]	12 [8, 16]

We estimated that the majority of the study population experienced high levels of anxiety (64.6%, [95% CI: 58.9, 70.2]) with females being in the “high levels of anxiety” category more frequently than males (72.5% [95% CI: 66.1, 78.9] vs 55.9% [95% CI: 46.6, 65.1]), while 62.6% [95% CI: 57.2, 67.9] were estimated to have high sleep efficacy (73.2% [95% CI: 65.6, 80.9%] of the males and 53.3% [95% CI: 46.3, 60.3] of the females). Over 50% were estimated to be in good or very good health with no differentiation between males and females, 42% did not have any chronic condition and less than 5% reported more than three (Table S4). The most commonly reported chronic disease was musculoskeletal problems followed by allergies. Overall, 28.6% [95% CI: 23.5, 33.6] were estimated to have two or more chronic diseases. Twenty-two percent of the study population reported that they had done the flu vaccine. The frequency of those reporting having done the flu vaccine at least once was higher in the age groups 60–69 and 70+ (Table S5).

#### Specific external exposome domain

##### Lifestyle and habits (during the measures)

We estimated that the majority of the population were non-smokers (71.3% [95% CI: 66.3, 76.4]) and 41.4% [95% CI: 35.9, 47] did not consume alcohol (Table S6). Moreover, 24.2% [95% CI: 19.3, 29.1] exercised for at least 30 min 2–3 times per week, 67.8% [95% CI: 63.3, 72.3] ate breakfast daily and 50.8% [95% CI: 45.6, 56] never consumed food not prepared at home (e.g., take way). Similar consumption patterns for fruits, meat and sugar in terms of portions consumed per day were estimated for males and females (Table S6). However, a difference was noted in the consumption of vegetables with females having an estimated median of two portions per day compared to one portion per day for the males (Table S6). With regards to personal hygiene and cleaning activities, 46.1% [95% CI: 40.5, 51.8] were estimated to have been washing their hands using soap more than 7 times per day (51.4% [95% CI: 45.1, 57.7] for the females and 40.4% [95% CI: 30.9, 50] for the males) and 18% [95% CI: 13.8, 22.1] using hand sanitizer with the same frequency. The median weekly number of times of bathroom cleaning was estimated to be 3 [interquartile range: 1–6] and it was higher for females compared to males (4 vs 3). Overall, the most frequently performed cleaning activity was cleaning the kitchen (median weekly number of times 7) while the median number of times of cleaning other house areas, i.e., bathroom and floors or any other cleaning activity was 3 and 2 times per week, respectively (Table S6). All cleaning activities were estimated to be more frequently performed by females compared to males.

##### Screen time and online communication during the measures

**Fig. 2:**
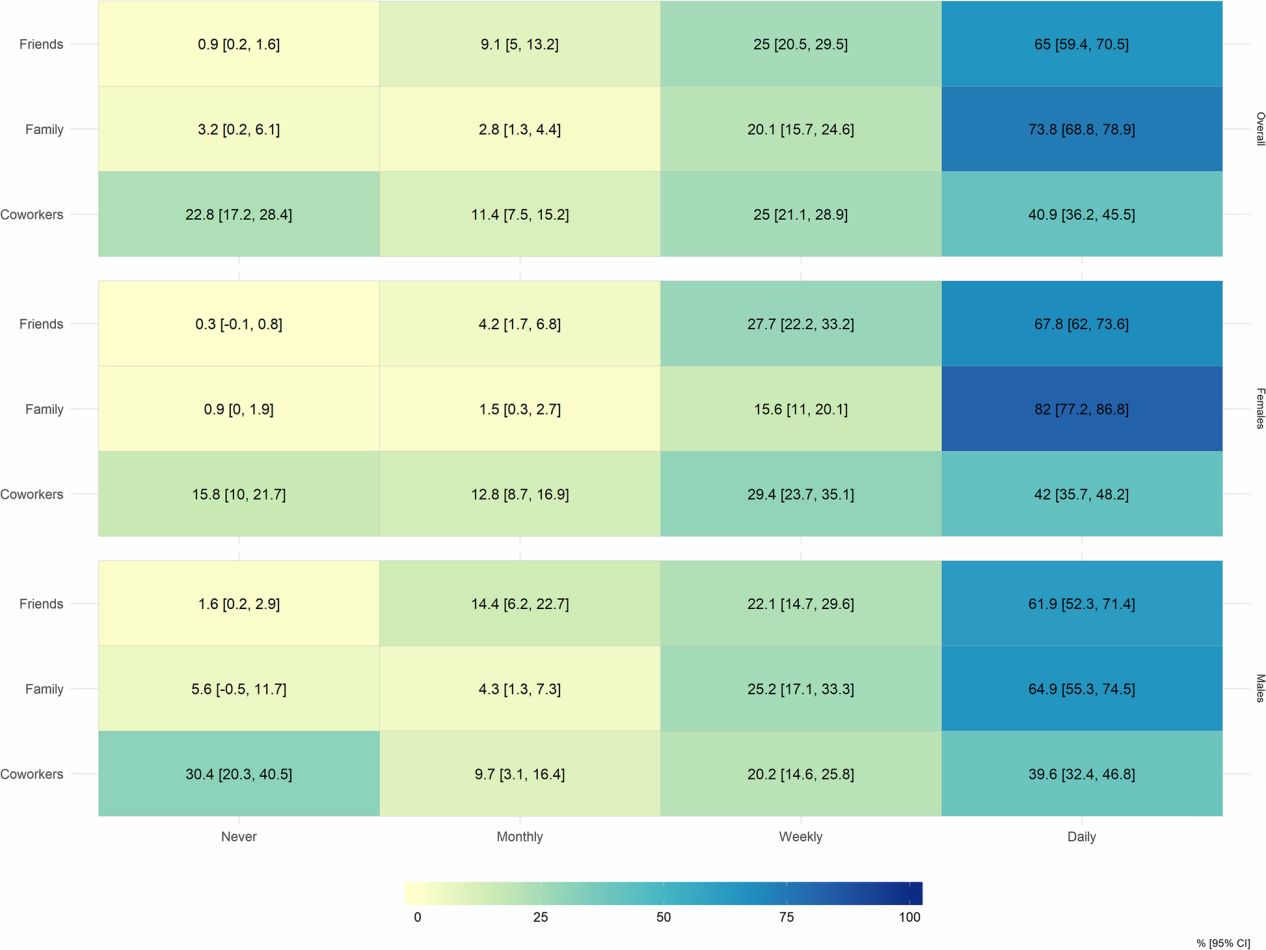
Phone and online communication with friends, family and coworkers, during the 2020 NPI population-wide measures, overall and by sex. The heatmap shows the estimated proportion [%, 95% CI] of the population communicating with friends, family and coworkers daily, weekly (at least once per week), monthly or never using the phone or online

With regards to screen time, 39% were estimated to be watching television or playing video games etc. for 4–7 h per day (Table S6). More than 50% of the study population were estimated to communicate via phone or using online platforms with friends and family daily and overall, 41% reported daily phone/online communication with coworkers (Fig. 2).

#### General external exposome domain

Most of the respondents reported living in the district of Limassol (42%). After weighting, the distribution of the study population resembled the one of the Cypriot population as estimated for 2019, i.e., 28.6% [95% CI: 27.8, 29.4] for Limassol, 39.6% [95% CI: 38.5, 40.7] for Nicosia, 15.4% [95% CI: 13.1, 17.7] for Larnaka, 10.8% [95% CI: 10.5, 11.1] for Paphos and 5.6% [95% CI: 5.4, 5.7] forrom Famagusta (Table 3). Moreover, 71.1% [95% CI: 66.3, 75.9] were estimated to be employed, 78.8% [95% CI: 74.1, 83.5] to having completed university and 60.8% [95% CI: 56.8, 64.9] to be married (Table 3). The distribution of these three characteristics (i.e., professional status, education and marital status) in the study population was not significantly modified after weighting (Table 3).

**Table 3:** General external exposome domain characteristics of the study population (weighted and unweighted)

	**Weighted** **% [95% CI]**			**Unweighted** **n (%)**		
	Overall	Females	Males	Overall	Females	Males
	594	311	283	594	369	225
**District**						
Limassol	28.6 [27.8, 29.4]	25.8 [19.4, 32.1]	31.7 [25.6, 37.8]	247 (42)	151 (41)	96 (43)
Nicosia	39.6 [38.5, 40.7]	39.3 [33, 45.7]	39.9 [33.8, 46]	238 (40)	147 (40)	91 (40)
Larnaka	15.4 [13.1, 17.7]	18.3 [11.1, 25.4]	12.3 [8.1, 16.5]	54 (9)	37 (10)	17 (8)
Paphos	10.8 [10.5, 11.1]	10.2 [5.9, 14.5]	11.5 [6.8, 16.1]	28 (5)	16 (4)	12 (5)
Famagusta	5.6 [5.4, 5.7]	6.4 [4.1, 8.8]	4.6 [2, 7.2]	27 (5)	18 (5)	9 (4)
**Education level**						
University (at least bachelor’s)	78.8 [74.1, 83.5]	79.1 [74.2, 84.1]	78.4 [70.1, 86.8]	478 (80)	298 (81)	180 (80)
Secondary/post-secondary (non-tertiary/non-university)	21.2 [16.5, 25.9]	20.9 [15.9, 25.8]	21.6 [13.2, 29.9]	116 (20)	71 (19)	45 (20)
**Professional status**						
Employed	71.1 [66.3, 75.9]	75.9 [70.2, 81.7]	65.8 [57.2, 74.4]	480 (81)	294 (80)	186 (83)
Student	9.5 [7.4, 11.7]	14.4 [10.6, 18.2]	4.2 [1.8, 6.6]	62 (10)	49 (13)	13 (6)
Unemployed/stay-at-home	6 [3.7, 8.4]	5.8 [2.9, 8.6]	6.3 [2.5, 10.1]	33 (6)	21 (6)	12 (5)
Retired	13.4 [9.4, 17.3]	3.9 [-0.1, 8]	23.7 [15.2, 32.2]	19 (3)	5 (1)	14 (6)
**Marital status**						
Married/divorced/widowed	60.8 [56.8, 64.9]	57.2 [52.2, 62.2]	64.8 [57.1, 72.5]	343 (58)	207 (56)	136 (60)
Single/not married (in a relationship)	39.2 [35.1, 43.2]	42.8 [37.8, 47.8]	35.2 [27.5, 42.9]	251 (42)	162 (44)	89 (40)

### Indicators of compliance to non-pharmaceutical interventions (March–April 2020)

**Fig. 3:**
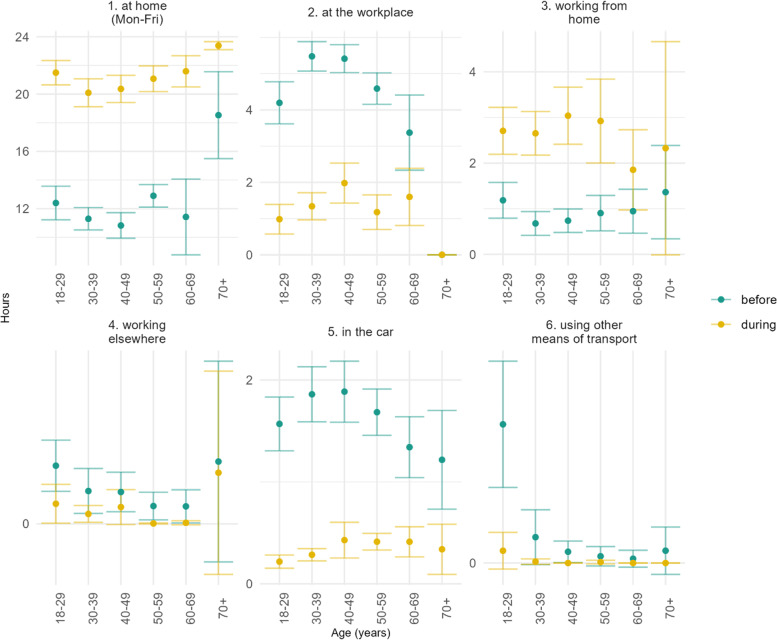
Estimated mean (and 95% CI) of the number hours per day spent in different settings before and during the spring 2020 population-wide measures in Cyprus by age group

Overall, an increase was noted in the number of hours spent at home and working from home and a decrease in the time spent at the workplace, working elsewhere and in the car or in other means of transportation (Fig. 3).

Overall, the estimated median number of contacts at home, at work, and elsewhere decreased during the measures compared to before (Table 4). The median number of contacts at work before the measures was 1.2, while during the implementation of the pandemic measures it dropped to 0 with the interquartile range (IQR) between 0 and 1. The median number of contacts at home decreased to a smaller extent, from 3 (IQR [1, 15]) to 2 (IQR [1, 3]). There was also a decrease in the number of contacts elsewhere from a median of 8 (IQR [5-18]) to 1 (IQR [0, 3]) before and during the measures, respectively. The decrease in the number of contacts was more evident at the workplace or elsewhere. Comparing the median decrease between males and females in the different places, a common trend was observed, i.e., higher decrease for the contacts at work or elsewhere, compared to home. However, it was observed that the change and the difference in the contacts at work and elsewhere was less for males compared to the median number of contacts in these places reported for females (Table 4).

**Table 4:** Estimated (weighted) number of contacts in different places before and during the spring 2020 pandemic measures in Cyprus by sex

	**Overall**	**Females**	**Males**
	594	311	283
**Before number of contacts home (median [IQR])**	3 [1, 5]	3 [2, 5]	3 [1, 5]
**During number of contacts home (median [IQR])**	2 [1, 3]	2 [1, 3]	1 [1, 3]
**Before number of contacts work (median [IQR])**	12 [3, 30]	15 [6, 40]	10 [0, 25]
**During number of contacts work (median [IQR])**	0 [0, 1]	0 [0, 0]	0 [0, 2]
**Before number of contacts elsewhere (median [IQR])**	8 [5, 18]	10 [5, 15]	8 [4, 20]
**During number of contacts elsewhere (median [IQR])**	1 [0, 3]	1 [0, 3]	1 [0, 4]
**Percent change number of contacts home (median [IQR])**	0 [-65.7, 0]	0 [-50, 0]	0 [-66.7, 0]
**Percent change number of contacts work (median [IQR])**	-100 [-100, -49.9]	-100 [-100, -70]	-90 [-100, 0]
**Percent change number of contacts elsewhere (median [IQR])**	-85.5 [-100, -60]	-90 [-100, -70]	-80 [-100, -50]
**Difference (before-during) number of contacts home (median [IQR])**	0 [-2, 0]	0 [-2, 0]	0 [-2, 0]
**Difference (before-during) number of contacts work (median [IQR])**	-10 [-30, -2]	-15 [-30, -5]	-8 [-21.5, 0]
**Difference (before-during) number of contacts elsewhere (median [IQR])**	-6 [-14, -3]	-7 [-13, -4]	-5 [-15, -2]

**Fig. 4:**
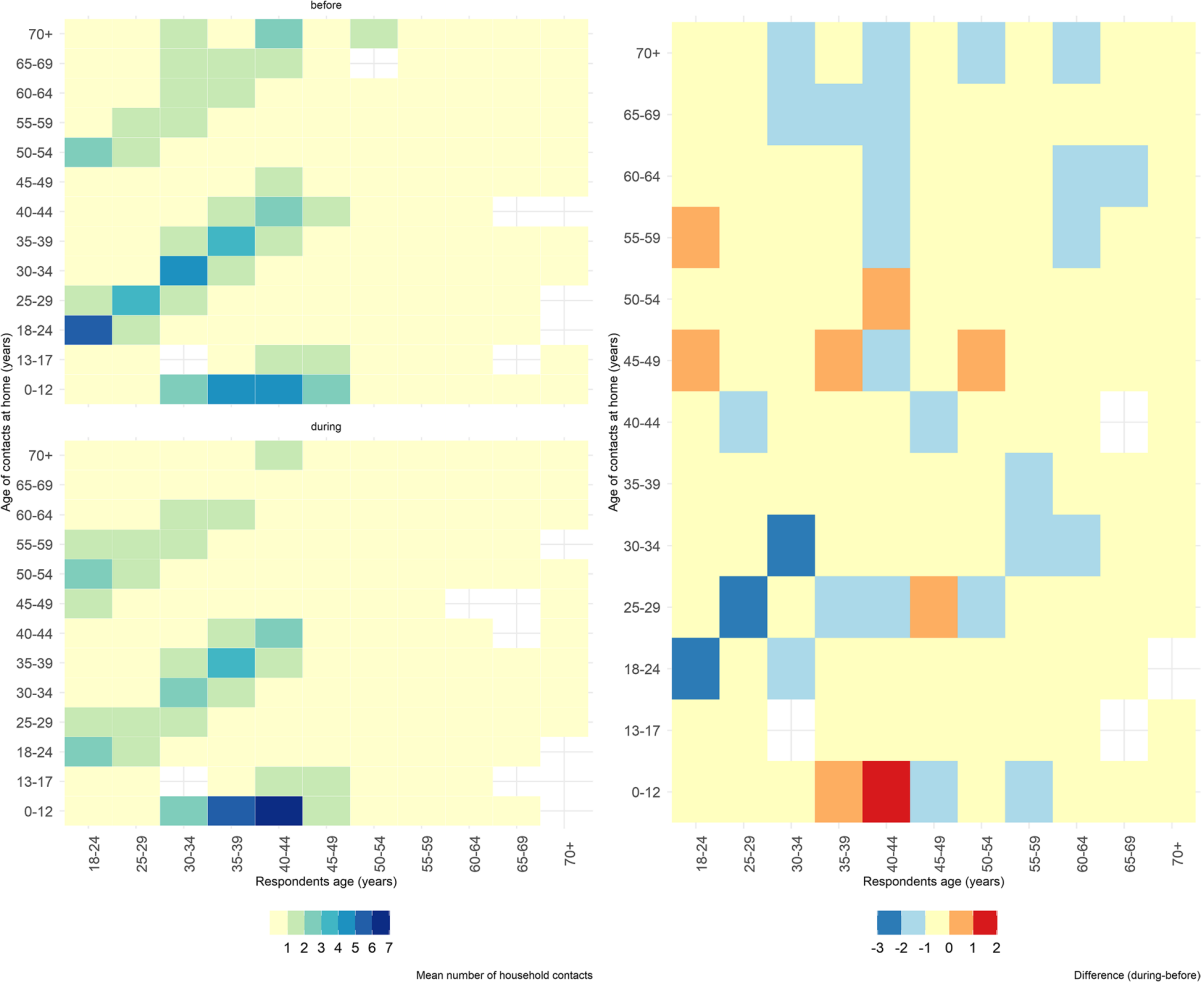
Average number of contacts before and during the spring 2020 population-wide measures in Cyprus (left) and difference in the number of contacts before and during the measures by age group (right)

The decrease in the number of contacts was also evident in the heatmaps that showed the mean number of contacts per age group (Fig. 4). Before the measures, respondents aged 18–24 year-olds reported higher average number of household contacts with persons in the same age group (average of 6). During the measures, the average number of household contacts of 18–24 year-olds with other 18–24 year-olds dropped to three (Fig. 4). During the measures, the contacts between 40–44 year-olds and 0–12 year-olds increased from five to seven and the contacts between 35–39 year-olds and 0–12 year-olds from five to six. The number of contacts of respondents aged below 60 with contacts in older age groups (e.g., above 50 years) decreased during the measures (Fig. 4). The decrease in the number of contacts was also reflected in the number of vulnerable contacts of the respondents (Figure S1).

**Fig. 5:**
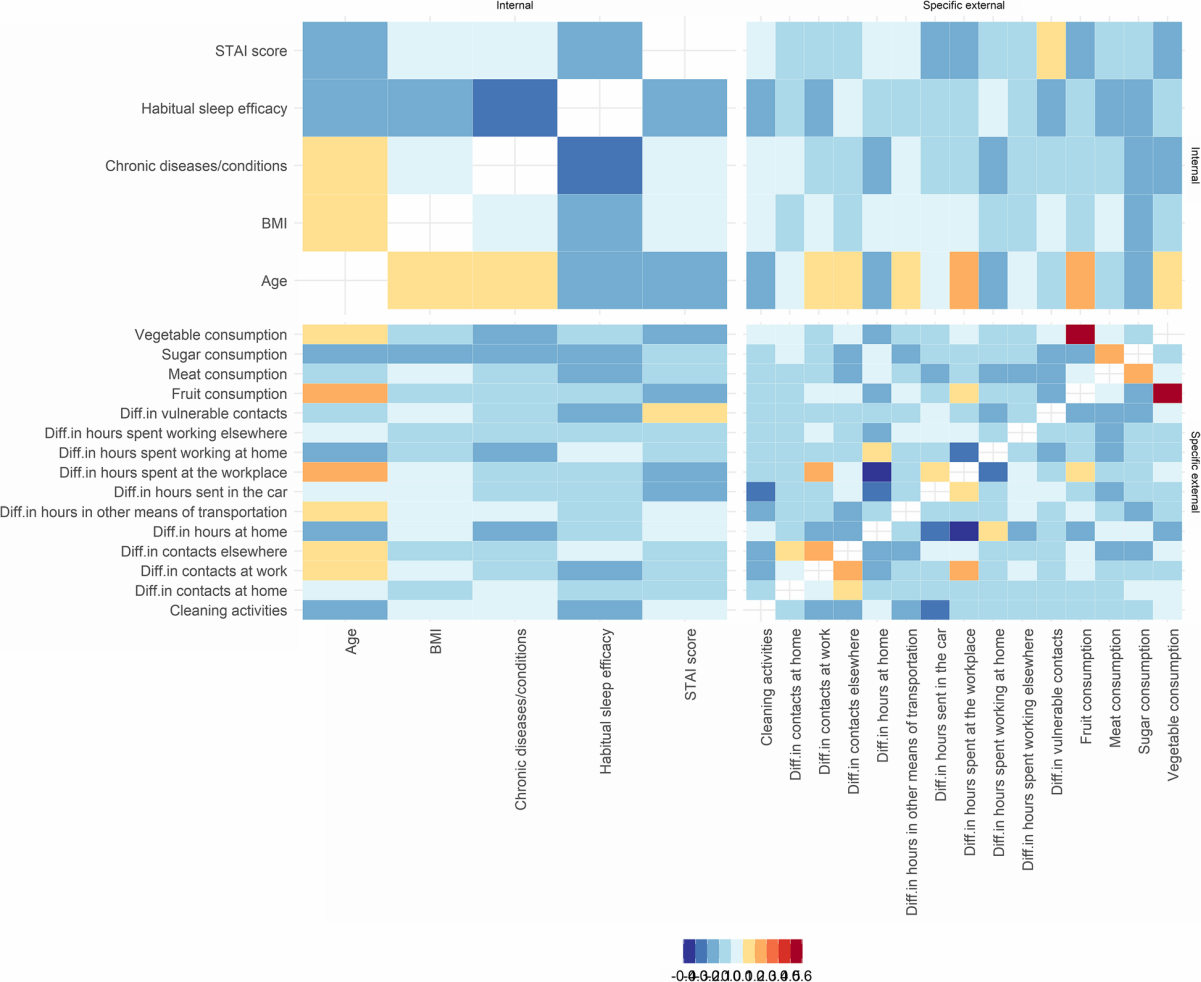
Heatmap of the weighted correlations between the different exposome parameters described in the Exposome@home study

In the weighted correlation analysis, among the variables of the specific external exposome, the highest positive correlation was observed between the consumption of fruits and vegetables (*r* = 0.6), while among the variables of the internal exposome, the highest correlation was observed between the number of chronic diseases/conditions and age, and BMI and age (r = 0.2). Negative correlations among the specific external variables were more evident between the difference in the hours spent at the workplace and the difference in the hours spent at home (before and during the measures, *r* = -0.4). With regards to the internal exposome, the highest negative correlation was observed between sleep efficacy and the number of chronic diseases/conditions (*r* = -0.2) (Fig. 5).

#### Exposome-wide association study (ExWAS)

In the ExWAS regression analysis, after adjusting for age and sex, increased hours spent at home during the period of implemented COVID-19 measures was associated with increased number of hours spent at work before the measures (OR 1.38, 95% CI [1.19, 1.60]) and never communicating with the coworkers via phone or online compared to communicating with the coworkers daily (OR 0.28, 95% CI [0.132, 0.598]). The number of hours spent at home during the measures was associated with the number of hours spent at the workplace before the measures (β= -0.87, 95% CI [-1.21,-0.53]), with being retired vs being currently employed (β= 2.32, 95% CI [1.70, 2.93]), and with communication patterns, i.e., communicating with the coworkers using the phone or online platforms sometimes per month compared to every day (β= 2.38, 95% CI [1.31, 3.44]), and with having no communication with one’s family over social media vs communicating daily  (β= 2.16, 95% CI [1.16, 3.15]). All results of the ExWAS analysis can be found in the Supplementary Material along with the detailed output of the analysis.

### Transmission modeling on synthetic human proximity networks

**Fig. 6:**
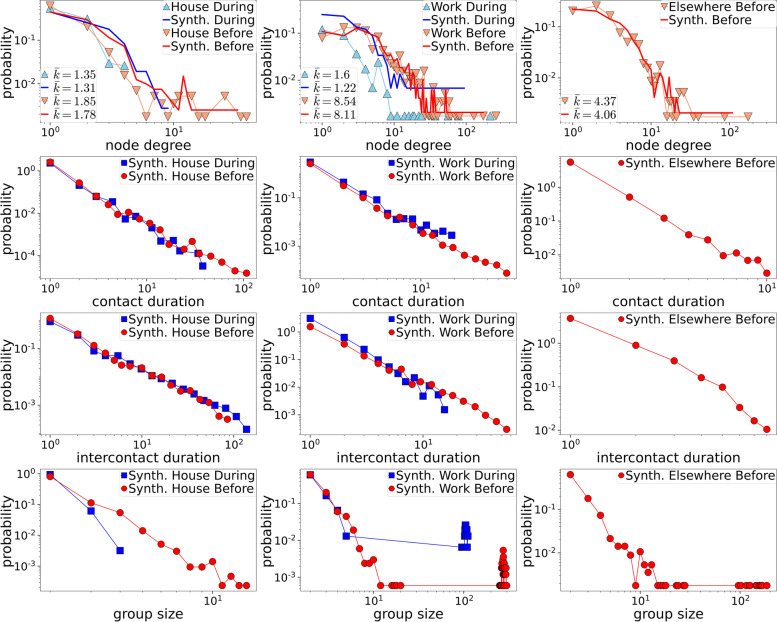
Properties of the synthetic human proximity networks generated with the dynamic-${\mathbb{S}}$ ^1^ model from the normalized number of contacts. First row: distribution of node degrees (i.e. number of contacts). The sky blue and light salmon triangles correspond to the real node degree distributions in the exposome data during and before the measures, respectively. The blue and red solid lines correspond to the synthetic human proximity networks during and before the measures, respectively. The legend in each plot also shows the average node degree $\overline{\mathbf{k} }$ in reality and in the synthetic networks. Second row: distribution of contact durations. Third row: distribution of intercontact durations. Fourth row: distribution of group sizes

In total, 578 respondents were included in the network analysis, after excluding those with missing number of contacts. The dynamic-${\mathbb{S}}$ 1 model adequately reproduced the real node degree distribution of the Exposome@home respondents, suggesting that the measures were successful in altering properties of the underlying contact network that are crucial for efficient transmission. In effect, node degrees, contact and intercontact durations and group size formations were reduced both for house and workplace contacts (Fig. 6). As anticipated, the average node degree (number of contacts per individual) over all time slots in each setting was approximately the same as the average reported number of contacts in the Exposome@home survey. Giant connected components in the elsewhere and workplace settings before the measures were formed in the networks generated from the non-normalized contacts (Figure S2). By contrast, after normalization, smaller connected components formed in all cases and giant connected components only appeared in the workplace setting before the measures (Fig. 6).

**Fig. 7:**
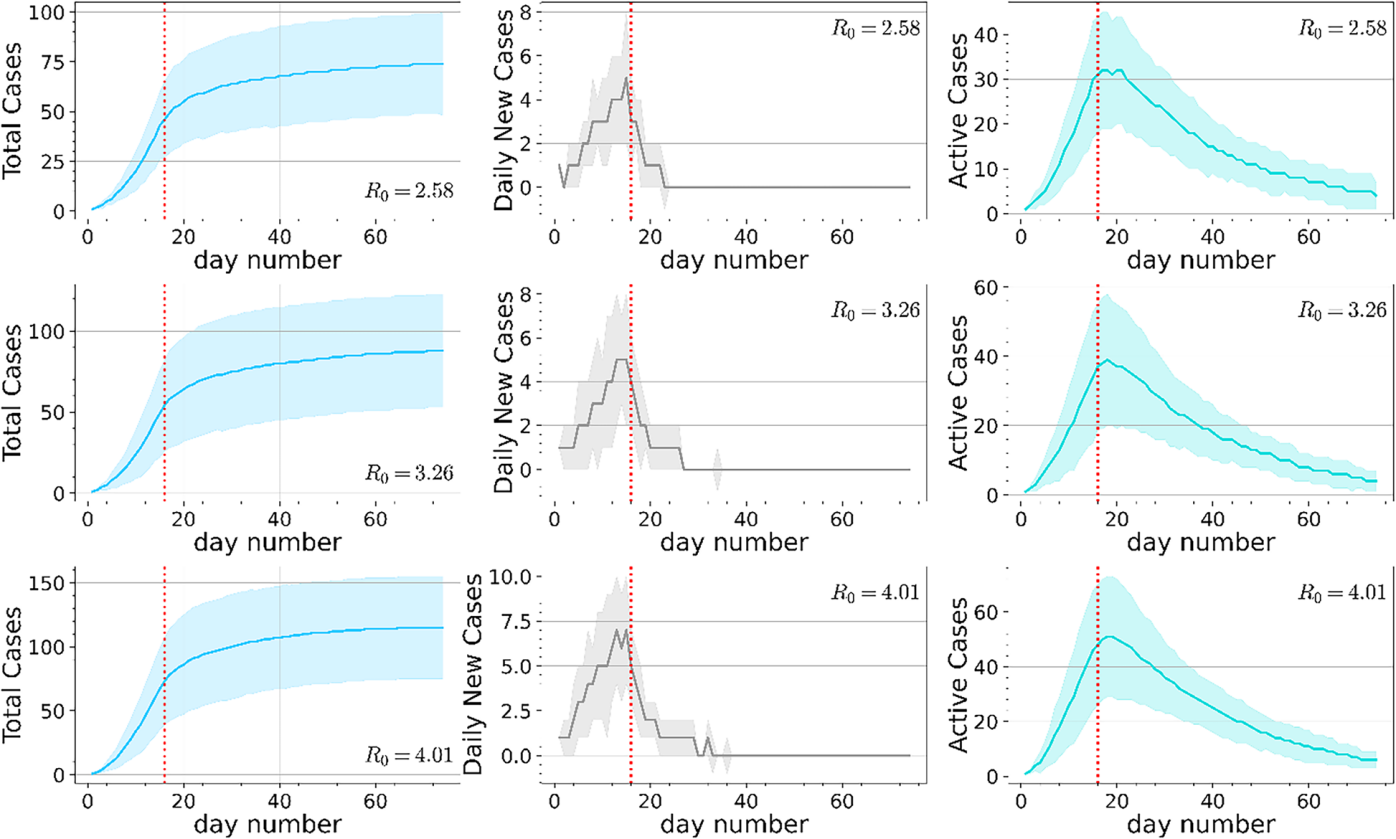
Dynamic SEIR simulation results on the synthetic human proximity networks generated with the dynamic-${\mathbb{S}}$ ^1^ model using the normalized number of contacts. Top row: total cases, daily new cases and active cases, averaged over 50 dynamic SEIR processes for R_0_ = 2.58. Middle and bottom rows show the same results as the top row but for R_0_ = 3.26 and R_0_ = 4.01, respectively. The shaded area in each plot corresponds to one standard deviation away from the average. The vertical red line marks the beginning of the measures

In Fig. 7 we plotted the total number of cases, the daily new cases and the active cases. The results are averages over 50 dynamic SEIR processes on the synthetic temporal networks of normalized contacts, for R0 = 2.58, 3.26 and 4.01. Results appeared qualitatively similar to the corresponding real trends observed from March 9 until May 21, 2020 in Cyprus [2], where the total cases began to stabilize and the daily new cases as well as the active cases dropped after the population-wide NPI measures were enforced (marked with the vertical red line). A similar pattern was observed in the synthetic temporal networks using non-normalized contacts, but a larger portion of the population became infected due to the formation of larger groups and due to the higher average degree than that in the normalized case (Figure S3).

**Fig. 8:**
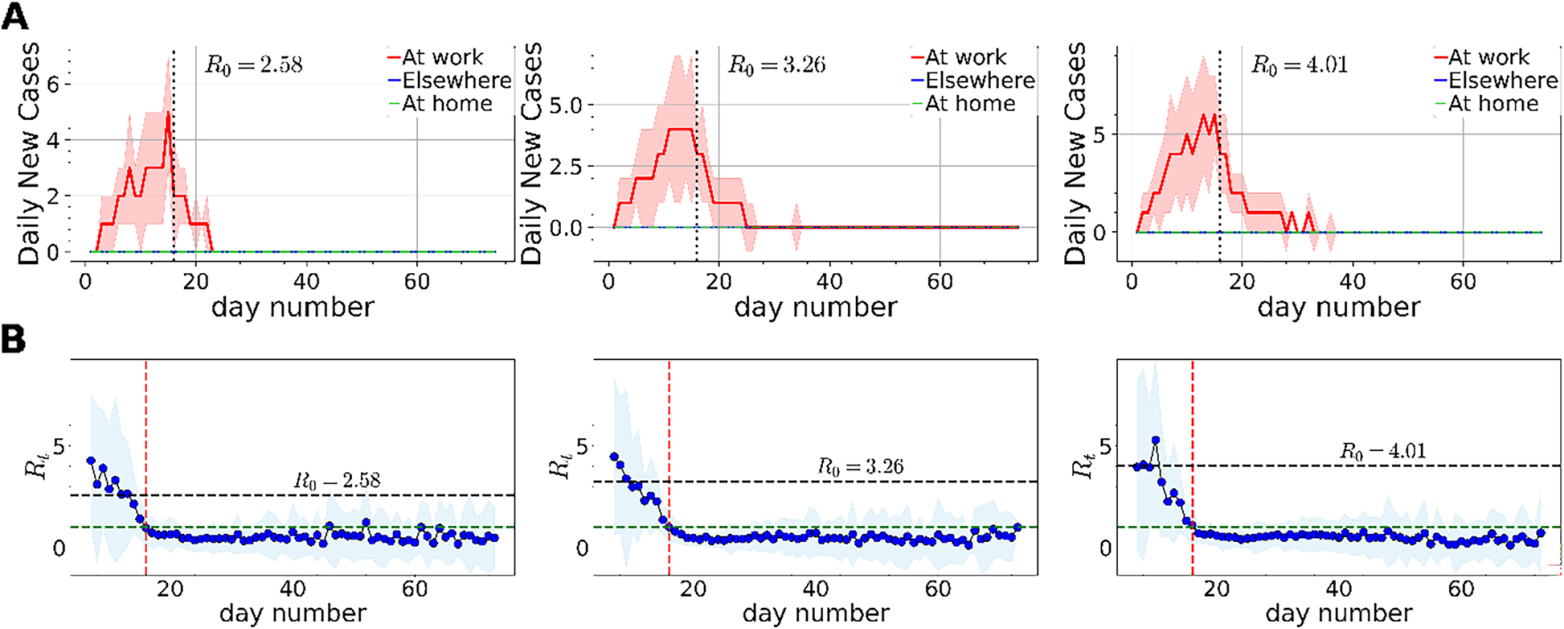
**A** Daily new cases originated at work (red lines), elsewhere (blue lines) and at home (dashed green lines), averaged over 50 dynamic SEIR processes on synthetic human proximity networks using the normalized number of contacts. Cases originated elsewhere and at home are approximately zero after averaging. The shaded areas in each plot correspond to one standard deviation away from the average. The vertical black line marks the beginning of the measures. **B** Evolution of R_t_ averaged over 50 dynamic SEIR processes on the synthetic human proximity networks using the normalized number of contacts. The shaded area in each plot corresponds to one standard deviation away from the average. The green horizontal line marks R_t_ = 1 whereas the horizontal black line marks the corresponding initial R_0_ value in the simulations. The vertical red line marks the beginning of the measures

The simulations indicate that the majority of the expected daily new cases for each setting (work, elsewhere or home) averaged over 50 dynamic SEIR process originated at work (Fig. 8A). Results for non-normalized contacts showed similar trends (Figure S4). The Rt dropped below one very close in time with the beginning of the NPI measures (red vertical line) and remained as is until the end of the simulations using either the normalized or raw data (Fig. 8B and Figure S5).

## Discussion

In this study, we described how the exposomic profile of Cyprus’ general population was shaped in the first wave of the COVID-19 epidemic (spring 2020), as a result of the population-wide measures and we estimated changes in a number of exposomic parameters compared to the period before the measures. During the first COVID-19 wave, in spring 2020, schools were closed, working from home was either mandatory or strongly encouraged and stay-at-home orders were implemented, leading to overall changes in people’s lifestyle. The median decrease in the number of contacts at home was the same for males and females. However, for the number of contacts at work, the decrease was higher for females, both in terms of percent change and in absolute numbers. Mobility and transport restrictions, tele-working and decrease in the number of contacts were observed. Daily communication using the phone or online platforms was reported by the majority of population, mostly with other family members and friends.

Compliance with the measures led to a drastic decrease in the number of contacts. Particularly in the age groups of 35–39 and 40–44 years old, there was a noted increase in the number of contacts with younger age groups i.e., 0–12 years, which might be the result of the increased number of hours spent at home. Positive correlation was also observed between the decreasing number of contacts at work and elsewhere and age. The time spent at work before the measures was associated with higher compliance to the measures, i.e., with higher number of hours spent at home during the measures. Additionally, there was a direct association noted between the number of hours spent at home during the measures and the number of hours spent at work before the measures, with the working status as well as with the use of communication patterns.

We estimated that the majority of the population daily used phone or online platforms for communication with friends and family, and had high anxiety levels. A previous study on elderly population showed association between loneliness and virtual communication in Brazil [31]. In Cyprus, the communication patterns during the pandemic have not been explored in depth. However, another cross-sectional study conducted also in spring 2020, showed that during the measures there was a higher risk for increased anxiety and depression [32].

Some eating habits, such as consuming vegetables and fruits were positively correlated between them. Changes in dietary habits were indicated by various surveys during the implementation of stay-at-home orders in the first pandemic wave; a decrease was noted in eating food prepared outside home in a U.S. study [33]. In this Exposome@home|COVID-19 study, the majority reported never ordering food or eating food prepared not at home, consuming at least one portion of fruits and/or vegetables daily, exercising at least 2–3 times per week (for 30 min per day) and having breakfast daily. These habits were probably carried over from the habits before the measures and were reinforced when the population had more time at home to prepare meals, or exercise even when the gyms and sports facilities were closed. It should be noted that when the stay-at-home orders were in place, going out for exercise was allowed. Therefore, continuing outdoor exercising should not have been affected by the measures.

The study extended to the generation of contact networks to describe the impact of the measures in social contacts and to simulate SARS-CoV-2 transmission. Recent works considered classic static networks [28, 29] and real human proximity networks [34] to simulate the transmission of SARS-CoV-2 using compartmental models such as the SEIR model. Previous works have also considered synthetic human proximity networks to simulate epidemic processes [33, 34]; however, the generative models used in previous works produce networks with unrealistic properties. In contrast, in this work we construct synthetic human proximity networks using the recently proposed dynamic-S^1^ model, which has been shown to adequately reproduce the main properties of real human proximity systems.

The temporal network analysis showed that the synthetic human proximity networks generated with the dynamic-${\mathbb{S}}$ ^1^ model adequately reproduced the real distribution of the reported number of contacts from the Exposome@home survey. Furthermore, the SARS-CoV-2 transmission simulations presented in the main text captured the behavior observed in Cyprus, before and during the measures. Results of the temporal network analysis showed that the majority of the cases originated at work, in agreement with reports of large number of clusters of SARS-CoV-2 cases in the workplace across the EU and the UK [35]. Overall, these results suggest that this modeling approach can be a useful tool for studying hypothetical scenarios where the effect of different measures can be tested, either by changing the properties of the network and/or the parameters of the dynamic SEIR simulations, thus providing a theoretical justification in favor or against the implementation of specific NPI measures. Additionally, the analysis of the properties of the synthetic temporal networks generated by the model could help in identifying overestimations in the reported number of contacts in a survey, if unexpectedly large connected components are observed in the synthetic networks.

In this study, survey responses were used to describe the exposomic profile of the general population during the implemented NPI measures and to accordingly model human contact networks. This approach aimed to generate a comprehensive profile of the population in a period where social norms were modified due to the pandemic and had several limitations and strengths. Data collection was conducted online which might have led to overrepresentation of population with access to the internet and those who can make use of an online questionnaire. Additionally, the short time period during which the questionnaire was available might have hindered a higher sample size. To overcome this limitation, all estimates were based on weighting of the study population, using the raking method and using the most recent population estimates by age group, sex, and district. A recall bias associated with the retrospective self-reporting of pre-pandemic habits is possible. Taking into consideration these limitations, as well as the fact that this was a cross-sectional study, this study allowed us to describe external and internal exposome parameters and assess the compliance to the measures implemented in spring 2020. After spring 2020, and following the epidemiological situation, different measures were implemented in Cyprus including additional school closures, stay-at-home orders, mask wearing mandates. The epidemiologic situation and the escalation/de-escalation of the measures is expected to have modified the lifestyle of individuals, the number and frequency of social contacts, risk perceptions etc. This adds weight to the contribution of the external exposome parameters in shaping up the exposomic profile of both individuals and populations.

Population-wide NPI measures taken to curb the COVID-19 pandemic have influenced all aspects of life, but the degree and the extent of such lifestyle and behavioral changes in people’s daily routine warrants further study of their possible longer-term effects and whether such effects on the exposomic profile would be reversible or not. The exposome concept allowed for the comprehensive consideration of relevant exposome variables that capture a large part of daily lifestyle and routine. The knowledge generated from this survey can be used to inform about the exposomic profile of a population of interest and become the baseline for future monitoring of the population’s exposomic profile alterations. It is warranted that exposomic tools may be integrated in infectious diseases surveillance/monitoring systems, while the exposome utility could aid in risk assessment, preparedness and response to events of wider public health impact [17]. Monitoring the exposomic profile of the general population at times of implementing a suite of NPI measures over a longer period of time would reveal trends and patterns of interest to epidemic intelligence or to chronic disease monitoring systems. The implications of this project are important, because the future implementation of population-wide measures for infectious disease control and prevention shall be systematically monitored using the exposome concept to allow for the efficient spatiotemporal evaluation of population health indicators associated with such intervention measures. More studies are needed to describe the longer-term impact of NPI-based pandemic response measures on key population health indicators.

## Supplementary Information

**Additional file 1.** Supplementary Information.

## Data Availability

The datasets supporting the conclusions of this article are included within the article (and its Additional files). The input data, scripts and output for the main statistical analysis is available upon request from the corresponding author (KCM). The code implementing the dynamic-S1 model along with scripts for computing various temporal network properties are available at https://bitbucket.org/fpapadop/dynamic-s1/src/master/. The code implementing the dynamic SEIR process is available at https://bitbucket.org/mrodrflr/dynamic-seir/src/master/
