# Supplementary material for: Population-wide measures due to the COVID-19 pandemic and exposome changes in the general population of Cyprus in March–May 2020
Source: BMC Public Health. 2022 Dec 6;22:2279. doi: 10.1186/s12889-022-14468-z (PMC9724426; doi:10.1186/s12889-022-14468-z)
Supplement: Supplementary file 1 — Additional file 1. Supplementary Information. [file 12889_2022_14468_MOESM1_ESM.pdf]

# Supplementary Information

## Population-wide measures due to the COVID-19 pandemic and exposome changes in the general population of Cyprus in March-May 2020

Xanthi D. Andrianou[1], Corina Konstantinou[1], Marco A. Rodríguez-Flores[2], Fragkiskos Papadopoulos[2], Konstantinos C. Makris[1]*

[1] Cyprus International Institute for Environmental and Public Health, Cyprus University of Technology, Limassol, Cyprus

[2] Department of Electrical and Computer Engineering, Cyprus University of Technology, Limassol, Cyprus

# Tables

**Table S 1** Population estimates for 2019 per district, sex and age group for the population in the government-controlled areas of the Republic of Cyprus according to the Cyprus Statistical Services used in weighting the study population (Source: https://www.cystat.gov.cy/el/PublicationList?s=46).

| | **Population estimates 2019** |
|---|---|
| **District** | |
| **Nicosia** | 346400 (39) |
| **Famagusta** | 48900 (6) |
| **Larnaka** | 149000 (17) |
| **Limassol** | 248300 (28) |
| **Paphos** | 95400 (11) |
| | |
| **Age group** | |
| **18-29** | 158740 (22) |
| **30-39** | 143600 (20) |
| **40-49** | 111400 (16) |
| **50-59** | 107600 (15) |
| **60-69** | 94600 (13) |
| **70+** | 99900 (14) |
| | |
| **Sex** | |
| **Males** | 346860 (48) |
| **Females** | 368980 (52) |

**Table S 2** Summary of the number of responses on the variables about the number of contacts imputed or not to the 99th percentile of the respective respondents

| Period | Place | p99 | | n | % |
|---|---|---|---|---|---|
| Before | Elsewhere | 100 | Imputed to 100 | 5 | 0.8 |
| Before | Elsewhere | | Not imputed | 592 | 99.2 |
| Before | Home | 80 | Imputed to 80 | 5 | 0.8 |
| Before | Home | | Not imputed | 592 | 99.2 |
| Before | Work | 277 | Imputed to 277 | 6 | 1 |
| Before | Work | | Not imputed | 591 | 99 |
| During | Elsewhere | 20 | Imputed to 20 | 1 | 0.2 |
| During | Elsewhere | | Not imputed | 596 | 99.8 |
| During | Home | 7 | Imputed to 7 | 3 | 0.5 |
| During | Home | | Not imputed | 594 | 99.5 |
| During | Work | 51 | Imputed to 51 | 6 | 1 |
| During | Work | | Not imputed | 591 | 99 |

**Table S 3** Internal exposome domain characteristics (unweighted) of the respondents to the questionnaire of the Exposome@home|COVID-19 study, Cyprus, 2020.

| | | | Unweighted | |
|---|---|---|---|---|
| | | **Overall** | **Females** | **Males** |
| **n** | | 594 | 369 | 225 |
| **Age group (%)** | **18-29** | 138 (23) | 105 (28) | 33 (15) |
| | **30-39** | 186 (31) | 117 (32) | 69 (31) |
| | **40-49** | 146 (25) | 83 (22) | 63 (28) |
| | **50-59** | 77 (13) | 44 (12) | 33 (15) |
| | **60-69** | 35 (6) | 17 (5) | 18 (8) |
| | **70+** | 12 (2) | 3 (1) | 9 (4) |
| **BMI (median [IQR])** | | 24.2 [21.4, 27.7] | 23.0 [20.7, 26.4] | 26.0 [23.5, 28.4] |
| **STAI score category** | **High anxiety** | 402 (68) | 276 (75) | 126 (56) |
| | **Moderate anxiety** | 101 (17) | 60 (16) | 41 (18) |
| | **No or low anxiety** | 91 (15) | 33 (9) | 58 (26) |
| **Minutes to sleep score (%)** | **0 (<16 mins)** | 309 (52) | 162 (44) | 147 (65) |
| | **1 (16-30 mins)** | 66 (11) | 46 (12) | 20 (9) |
| | **2 (31-60 mins)** | 161 (27) | 121 (33) | 40 (18) |
| | **3 (>60 mins)** | 58 (10) | 40 (11) | 18 (8) |
| **Sleep duration (%)** | **0 (>7 hours)** | 390 (66) | 253 (69) | 137 (61) |
| | **1 (6-6.99 hours)** | 127 (21) | 67 (18) | 60 (27) |
| | **2 (5-5.99 hours)** | 54 (9) | 33 (9) | 21 (9) |
| | **3 (<5 hours)** | 23 (4) | 16 (4) | 7 (3) |
| **Habitual sleep efficacy score category (%)** | **0 (>=85)** | 319 (61) | 181 (57) | 136 (69) |
| | **1 (75-84)** | 110 (21) | 73 (23) | 37 (19) |
| | **2 (65-74)** | 51 (10) | 35 (11) | 15 (8) |
| | **3 (<65)** | 39 (8) | 31 (10) | 8 (4) |
| **Health status (%)** | | | | |
| | **Very good** | 365 (62) | 221 (60) | 144 (64) |
| | **Good** | 191 (32) | 123 (33) | 68 (30) |
| | **So and so** | 35 (6) | 24 (7) | 11 (5) |
| | **Bad** | 2 (0) | 1 (0) | 1 (0) |
| **Chronic diseases** | **1** | 188 (32) | 121 (33) | 67 (30) |
| | **2 or more** | 161 (27) | 113 (31) | 48 (21) |
| | **None** | 245 (41) | 135 (37) | 110 (49) |
| **Influenza vaccine (%)** | **No** | 384 (65) | 241 (66) | 143 (64) |
| | **Yes** | 115 (19) | 71 (19) | 44 (20) |
| | **I don’t know/I don’t remember/I’m not sure** | 92 (16) | 55 (15) | 37 (17) |

**Table S 4** Number of chronic diseases/conditions.

| | | Weighted<br>% [95% CI] | | | | Unweighted<br>n (%) | |
|---|---|---|---|---|---|---|---|
| | **Overall** | **Females** | **Males** | | **Overall** | **Females** | **Males** |
| **Number of chronic diseases** | 594 | 311 | 283 | | 594 | 369 | 225 |
| | | | | | | | |
| **0** | 41.9% [36.5, 47.4] | 36.9% [30.6, 43.2] | 47.5% [38.1, 56.9] | | 245 (41) | 135 (37) | 110 (49) |
| **1** | 29.5% [24.5, 34.5] | 32.1% [26.2, 37.9] | 26.7% [18.4, 34.9] | | 188 (32) | 121 (33) | 67 (30) |
| **2** | 16.4% [12.3, 20.5] | 16.4% [11.8, 21] | 16.5% [9.4, 23.6] | | 96 (16) | 63 (17) | 33 (15) |
| **3** | 7.2% [4, 10.5] | 9.8% [5.1, 14.6] | 4.4% [0.1, 8.7] | | 36 (6) | 29 (8) | 7 (3) |
| **4** | 3.3% [1.6, 4.9] | 3.1% [1.2, 5] | 3.4% [0.6, 6.3] | | 20 (3) | 14 (4) | 6 (3) |
| **5** | 0.9% [-0.1, 1.9] | 0.3% [-0.1, 0.7] | 1.6% [-0.6, 3.7] | | 4 (1) | 2 (1) | 2 (1) |
| **6** | 0.2% [-0.1, 0.6] | 0.4% [-0.2, 1.1] | | | 2 (0) | 2 (1) | 0 (0) |
| **7** | 0.2% [-0.1, 0.4] | 0.3% [-0.1, 0.7] | | | 2 (0) | 2 (1) | 0 (0) |
| **9** | 0.3% [-0.3, 1] | 0.6% [-0.6, 1.9] | | | 1 (0) | 1 (0) | 0 (0) |

**Table S 5** Flu vaccination patterns by age group.

| | **Overall** | **18-29** | **30-39** | **40-49** | **50-59** | **60-69** | **70+** |
|---|---|---|---|---|---|---|---|
| **Weighted (n)** | 594 | 135 | 121 | 95 | 93 | 79 | 72 |
| **Flu vaccination (%)** | | | | | | | |
| **No** | 378 (64) | 67 (50) | 78 (65) | 69 (73) | 72 (79) | 54 (69) | 38 (52) |
| **Yes** | 132 (22) | 16 (12) | 24 (20) | 17 (18) | 18 (20) | 23 (28) | 34 (48) |
| **I don't know/I don't remember/I'm not sure** | 81 (14) | 51 (38) | 19 (16) | 8 (9) | 1 (1) | 2 (3) | 0 (0) |
| | | | | | | | |
| **Unweighted** | **Overall** | **18-29** | **30-39** | **40-49** | **50-59** | **60-69** | **70+** |
| **Flu vaccination (%)** | 594 | 138 | 186 | 146 | 77 | 35 | 12 |
| **No** | 384 (65) | 66 (48) | 119 (64) | 111 (76) | 60 (79) | 22 (63) | 6 (50) |
| **Yes** | 115 (19) | 19 (14) | 38 (21) | 25 (17) | 15 (20) | 12 (34) | 6 (50) |
| **I don't know/I don't remember/I'm not sure** | 92 (16) | 52 (38) | 28 (15) | 10 (7) | 1 (1) | 1 (3) | 0 (0) |

**Table S 6** Specific external exposome components asked in the survey (weighted and unweighted) overall and by sex.

| | | Weighted % [95% CI] | | | | Unweighted n (%) | |
|---|---|---|---|---|---|---|---|
| | **Overall** | **Females** | **Males** | | **Overall** | **Females** | **Males** |
| | 594 | 311 | 283 | | | | |
| **Smoking** | | | | | | | |
| **No** | 71.3% [66.3, 76.4] | 74.6% [68.8, 80.4] | 67.8% [59.4, 76.2] | | 423 (71) | 278 (76) | 145 (64) |
| **Yes, every day** | 24.4% [19.5, 29.3] | 20.8% [15.2, 26.4] | 28.3% [20.1, 36.6] | | 134 (23) | 70 (19) | 64 (28) |
| **Yes, occasionally** | 4.2% [2.6, 5.9] | 4.6% [2.1, 7.1] | 3.9% [1.8, 5.9] | | 35 (6) | 19 (5) | 16 (7) |
| **Frequency of alcohol consumption** | | | | | | | |
| **I don't drink alcohol** | 41.4% [35.9, 47] | 54.8% [48.3, 61.3] | 26.7% [18.5, 35] | | 252 (43) | 196 (53) | 56 (25) |
| **once per month** | 12.6% [9, 16.1] | 12.3% [8.5, 16.2] | 12.9% [6.8, 19] | | 84 (14) | 51 (14) | 33 (15) |
| **2-3 times per month** | 9.7% [6.3, 13] | 7.6% [4.6, 10.5] | 12.1% [5.8, 18.3] | | 59 (10) | 32 (9) | 27 (12) |
| **1-2 times per week** | 17.7% [13, 22.4] | 15.9% [9.9, 22] | 19.6% [12.7, 26.5] | | 101 (17) | 49 (13) | 52 (23) |
| **3-4 times per week** | 7.9% [4.6, 11.2] | 4.8% [2, 7.7] | 11.4% [5.2, 17.5] | | 43 (7) | 17 (5) | 26 (12) |
| **5-6 times per week** | 1.1% [0.3, 1.9] | 0.8% [0.1, 1.5] | 1.5% [0, 3] | | 10 (2) | 5 (1) | 5 (2) |
| **Every day or almost every day** | 9.5% [5.3, 13.7] | 3.8% [1.8, 5.9] | 15.8% [7.6, 24.1] | | 43 (7) | 19 (5) | 24 (11) |
| **Frequency of exercising for at least 30 mins per day** | | | | | | | |
| **Never** | 15.7% [12.2, 19.1] | 17.8% [13.2, 22.3] | 13.3% [7.9, 18.8] | | 104 (17.5) | 68 (18) | 36 (16) |
| **2-3 times per month** | 9.1% [6.4, 11.8] | 7.8% [4.6, 11] | 10.6% [6, 15.1] | | 55 (9) | 31 (8) | 24 (11) |
| **Once per week** | 11.9% [9, 14.8] | 15.4% [11.1, 19.8] | 8% [4.3, 11.8] | | 83 (14) | 60 (16) | 23 (10) |
| **2-3 times per week** | 24.2% [19.3, 29.1] | 23.1% [17.7, 28.6] | 16.5% [8.9, 24.1] | | 142 (24) | 86 (23) | 56 (25) |
| **4-6 times per week** | 18.8% [14.6, 23] | 16.5% [12.1, 20.9] | 21.3% [13.9, 28.7] | | 120 (20) | 67 (18) | 53 (24) |
| **Every day** | 16.7% [12.2, 21.2] | 16.9% [10.9, 22.8] | 16.7% [12.2, 21.2] | | 77 (13) | 48 (13) | 29 (13) |
| **I don't exercise due to health issues** | 1.1% [0.2, 2] | 1.9% [0.2, 3.5] | 0.2% [-0.2, 0.7] | | 7 (1) | 6 (2) | 1 (0.4) |
| **I don't exercise due to mobility issues** | 2.6% [-0.3, 5.6] | 0.7% [-0.1, 1.5] | 4.7% [-1.3, 10.8] | | 6 (1) | 3 (1) | 3 (1) |
| **Frequency of having breakfast** | | | | | | | |
| **Never** | 9.2% [6.1, 12.3] | 8.1% [4.5, 11.7] | 10.4% [5.3, 15.6] | | 51 (9) | 26 (7) | 25 (11) |
| **2-3 times per month** | 1.8% [0.4, 3.3] | 1.4% [-0.2, 3.1] | 2.3% [-0.1, 4.6] | | 9 (2) | 4 (1) | 5 (2) |
| **Once per week** | 4.4% [2.6, 6.1] | 3.6% [1.6, 5.6] | 5.2% [2.3, 8.2] | | 31 (5) | 15 (4) | 16 (7) |
| **2-3 times per week** | 10.8% [7.9, 13.8] | 13% [8.6, 17.4] | 8.5% [4.7, 12.2] | | 71 (12) | 48 (13) | 23 (10) |
| **4-6 times per week** | 5.9% [4.2, 7.7] | 5.9% [3.4, 8.4] | 5.9% [3.3, 8.6] | | 48 (8) | 26 (7) | 22 (10) |

| | | | | | | | |
|---|---|---|---|---|---|---|---|
| **Every day** | 67.8% [63.3, 72.3] | 68% [62.1, 73.8] | 67.7% [60.4, 74.9] | | 384 (65) | 250 (68) | 134 (60) |

**Table S 6 (cont.)** Specific external exposome components asked in the survey (weighted and unweighted) overall and by sex.

| | | Weighted % [95% CI] | | | | Unweighted n (%) | |
|---|---|---|---|---|---|---|---|
| | **Overall** | **Females** | **Males** | | **Overall** | **Females** | **Males** |
| | 594 | 311 | 283 | | | | |
| **Frequency of going out, ordering food** | | | | | | | |
| **Never** | 302 (51) | 185 (60) | 116 (41) | | 289 (49) | 197 (53) | 92 (41) |
| **2-3 times per month** | 23.3% [18.6, 28] | 22.8% [17.8, 27.9] | 23.8% [15.8, 31.8] | | 142 (24) | 87 (24) | 55 (24) |
| **once per week** | 16% [12.6, 19.4] | 12.8% [9.1, 16.4] | 19.5% [13.1, 25.9] | | 114 (19) | 64 (17) | 50 (22) |
| **2-3 times per week** | 7.1% [3.8, 10.4] | 4.2% [2, 6.3] | 10.3% [3.9, 16.8] | | 38 (6) | 18 (5) | 20 (9) |
| **4-6 times per week** | 1.7% [-0.1, 3.6] | 0.3% [-0.1, 0.8] | 3.3% [-0.6, 7.1] | | 8 (1.3) | 2 (0) | 6 (3) |
| **Every day** | 1.1% [-0.7, 2.8] | 0.2% [-0.2, 0.5] | 2.1% [-1.6, 5.7] | | 3 (0.5) | 1 (0) | 2 (1) |
| **Hand soap use (%)** | | | | | | | |
| **>7 times per day** | 46.1% [40.5, 51.8] | 51.4% [45.1, 57.7] | 40.4% [30.9, 50] | | 282 (47.5) | 193 (52) | 89 (39.6) |
| **4-7 times per day** | 42.4% [36.7, 48.1] | 41.7% [35.3, 48] | 43.2% [33.5, 52.9] | | 242 (41) | 149 (40) | 93 (41.3) |
| **1-3 times per day** | 11% [8, 13.9] | 6.7% [3.5, 9.8] | 15.7% [10.4, 21] | | 67 (11) | 25 (7) | 42 (18.7) |
| **<1 time per day** | 0.4% [-0.2, 1.1] | 0.2% [-0.2, 0.5] | 0.7% [-0.6, 2] | | 2 (0) | 1 (0) | 1 (0.4) |
| **Never** | 0.1% [-0.1, 0.2] | 0.1% [-0.1, 0.4] | | | 1 (0) | 1 (0) | 0 (0.0) |
| **Hand sanitizer use (%)** | | | | | | | |
| **>7 times per day** | 18% [13.8, 22.1] | 18.2% [13.4, 23.1] | 17.7% [10.5, 24.9] | | 99 (17) | 61 (17) | 38 (17) |
| **4-7 times per day** | 23% [19.1, 26.9] | 26.1% [20.4, 31.7] | 19.7% [14.3, 25.1] | | 153 (26) | 92 (25) | 61 (27) |
| **1-3 times per day** | 32.3% [27.2, 37.4] | 33% [27, 39] | 31.5% [22.7, 40.2] | | 199 (34) | 129 (35) | 70 (31) |
| **<1 time per day** | 18.8% [14.2, 23.4] | 16.2% [11.7, 20.8] | 21.6% [13.1, 30] | | 103 (17) | 65 (18) | 38 (17) |
| **Never** | 7.9% [4.5, 11.3] | 6.5% [2.3, 10.6] | 9.5% [3.3, 15.8] | | | | |
| **Screen time** | | | | | | | |
| **<1 hour per day** | 2.3% [0.7, 3.8] | 0.5% [-0.1, 1.1] | 4.2% [1, 7.3] | | 13 (2) | 2 (1) | 12 (4) |
| **1-3 hours per day** | 26.6% [21.7, 31.6] | 24% [18.5, 29.5] | 29.5% [20.8, 38.3] | | 158 (27) | 74 (24) | 84 (30) |
| **4-7 hours per day** | 42.3% [36.7, 47.8] | 42.3% [35.8, 48.8] | 42.2% [32.6, 51.8] | | 251 (42) | 131 (42) | 120 (42) |
| **8-11 hours per day** | 18% [14.8, 21.2] | 20.6% [15.7, 25.6] | 15.2% [10.7, 19.6] | | 107 (18) | 64 (21) | 43 (15) |
| **>11 hours per day** | 10.8% [7.7, 14] | 12.6% [8.4, 16.7] | 8.9% [4, 13.8] | | 64 (11) | 39 (13) | 25 (9) |

**Table S 6 (cont.)** Specific external exposome components asked in the survey (weighted and unweighted) overall and by sex.

| | | | Weighted | | | | Unweighted | |
|---|---|---|---|---|---|---|---|---|
| | | **Overall** | **Females** | **Males** | | **Overall** | **Females** | **Males** |
| **Food types consumption (portions per day) (median [IQR])** | | | | | | | | |
| | **Fruits** | 1 [1, 2] | 1.68 [1, 2] | 1 [1, 2] | | 1 [1, 2] | 1 [1, 2] | 1 [1, 2] |
| | **Vegetables** | 2 [1, 2] | 2 [1, 2] | 1 [1, 2] | | 2 [1, 2] | 2 [1, 2] | 1 [1, 2] |
| | **Meat** | 1 [0, 1] | 1 [0.36, 1] | 1 [0, 2] | | 1 [1, 1] | 1 [1, 1] | 1 [1, 2] |
| | **Sugar** | 1 [1, 2] | 1 [1, 2] | 1 [0.44, 2] | | 1 [1, 2] | 1 [1, 2] | 1 [1, 2] |
| **Cleaning (times/week) (median [IQR])** | | | | | | | | |
| | **Bathroom** | 3 [1, 6] | 4 [2, 7] | 2 [1, 5] | | 3 [1, 6] | 3 [2, 7] | 2 [1, 4] |
| | **Kitchen** | 7 [3, 7] | 7 [5, 7] | 5 [2, 7] | | 7 [3, 7] | 7 [5, 7] | 5 [2, 7] |
| | **Floor** | 3 [1, 5] | 3 [2, 6] | 2 [1, 4] | | 3 [1, 5] | 3 [2, 5] | 2 [1, 4] |
| | **Other cleaning** | 2 [1, 5] | 3 [1, 7] | 2 [1, 3.21] | | 3 [1, 5] | 3 [1, 6] | 2 [1, 4] |

**Table S 7** Average number of participants and average number of daily contacts per age group in the POLYMOD and exposome datasets. For the exposome data we consider contacts before and during the lockdown. Note that the participants in the exposome dataset are 18 years old and above. We assume that the 18- and 19-years old participants in the exposome dataset have similar contacts as the 15-19 years old age group in the POLYMOD dataset.

| Age group | Participants | | Average daily contacts | | |
|---|---|---|---|---|---|
| | POLYMOD | Exposome @home (phase A) | POLYMOD | Exposome@home before the measures | Exposome@home during the measures |
| 0-4 | 660 | 0 | 10.21 | N/A | N/A |
| 5-9 | 661 | 0 | 14.81 | N/A | N/A |
| 10-14 | 713 | 0 | 18.22 | N/A | N/A |
| 15-19 | 685 | 10 | 17.58 | 53.9 | 3.9 |
| 20-29 | 879 | 129 | 13.57 | 78.81 | 14.36 |
| 30-39 | 815 | 184 | 14.14 | 55.65 | 8.03 |
| 40-49 | 908 | 141 | 13.83 | 60.43 | 11.13 |
| 50-59 | 906 | 71 | 12.30 | 61.54 | 6.15 |
| 60-69 | 728 | 32 | 9.21 | 29.72 | 4.5 |
| 70+ | 270 | 11 | 6.89 | 9.27 | 3.36 |

# Figures

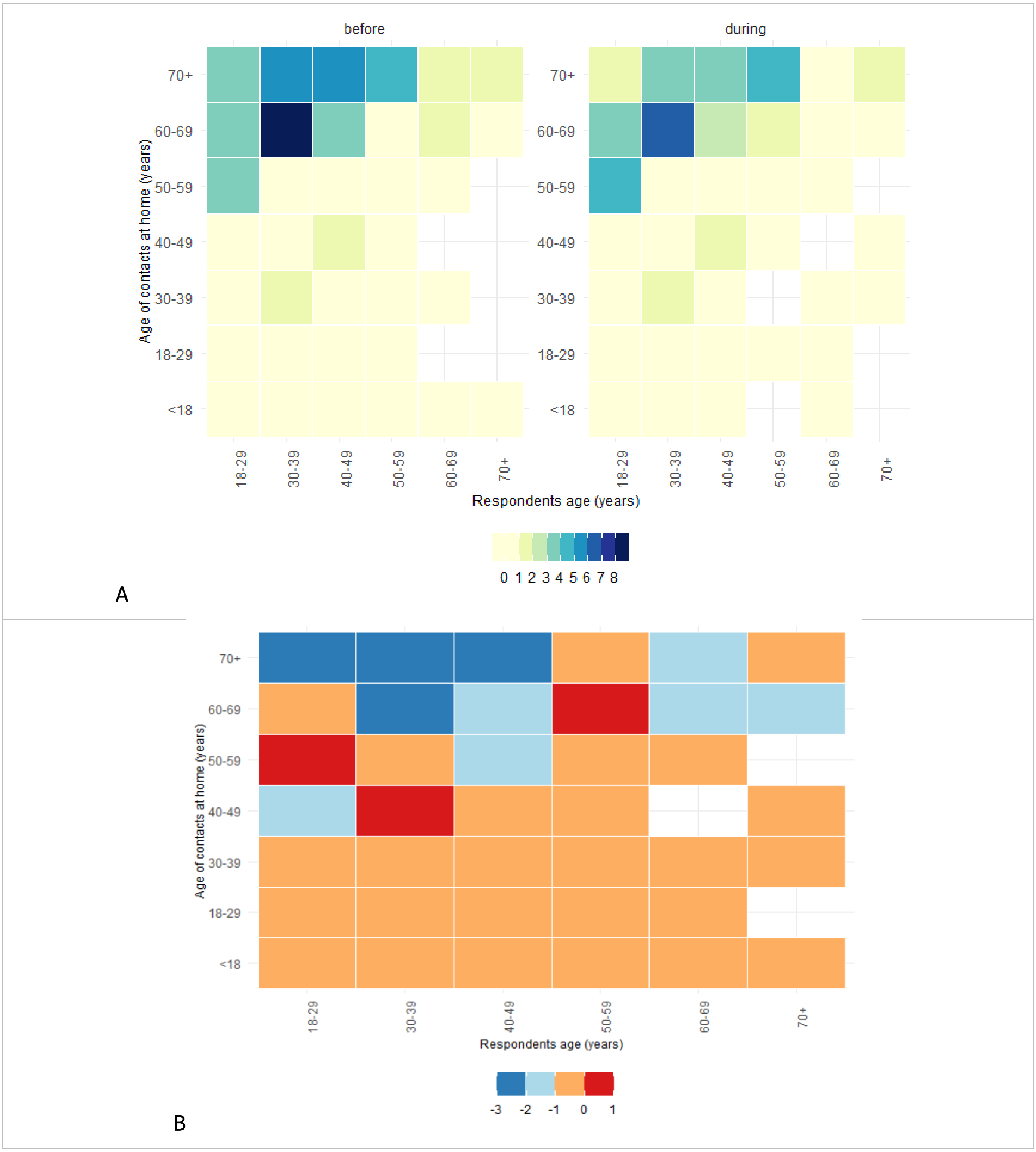


**Figure S 1 A.** Average number of contacts in vulnerable groups before and during the spring 2020 measures in Cyprus. B. Difference in the number of contacts in vulnerable groups before and during the measures by age group.

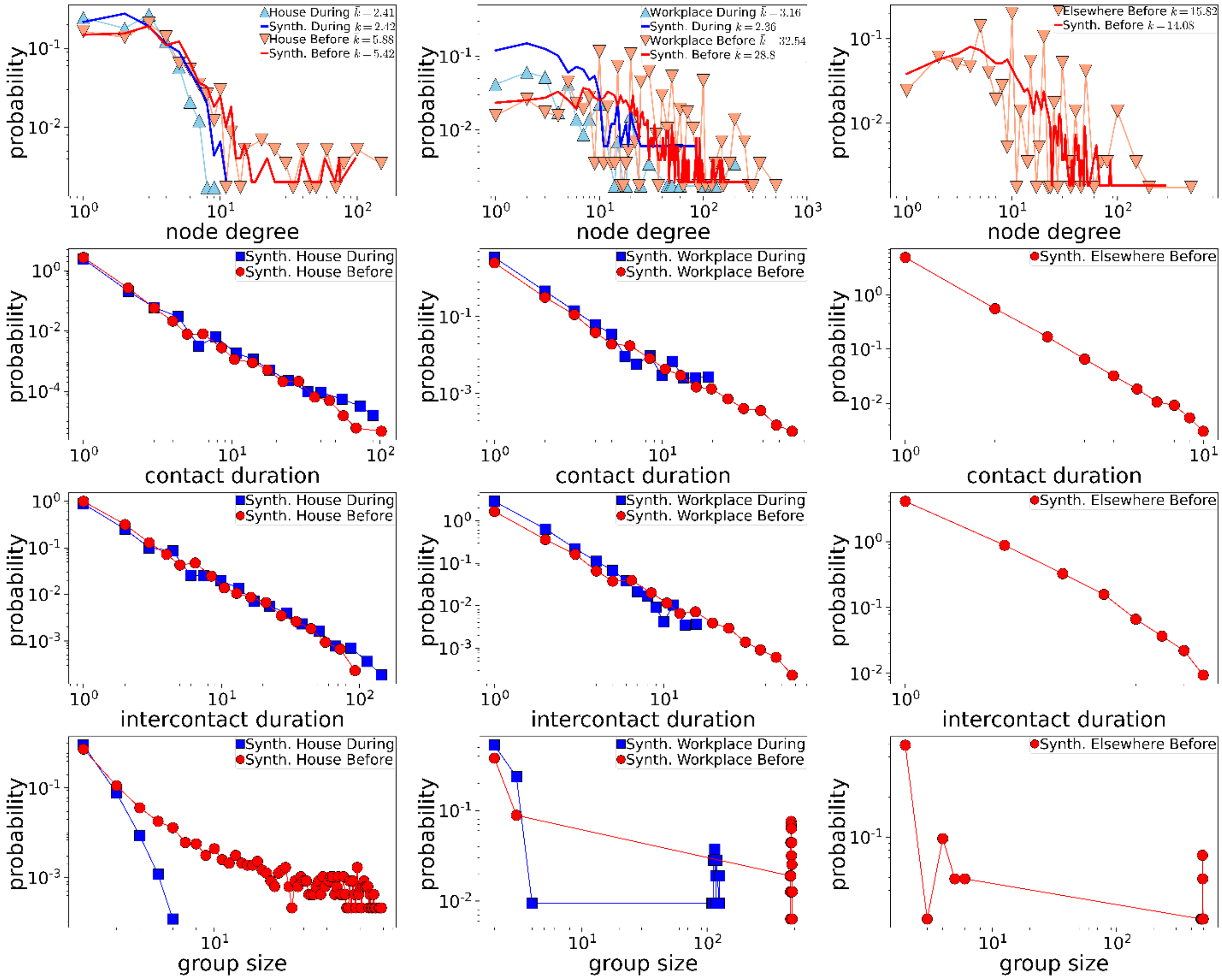


**Figure S 2** Properties of the synthetic human proximity networks generated with the dynamic-$\mathbb{S}^1$ model from the non-normalized number of contacts. First row: distribution of node degrees (i.e. number of contacts). The sky blue and light salmon triangles correspond to the real node degree distributions in the exposome data during and before the measures, respectively. The blue and red solid lines correspond to the synthetic human proximity networks during and before the measures, respectively. The legend in each plot also shows the average node degree $\bar{\mathbf{k}}$ in reality and in the synthetic networks. Second row: distribution of contact durations. Third row: distribution of intercontact durations. Fourth row: distribution of group sizes.

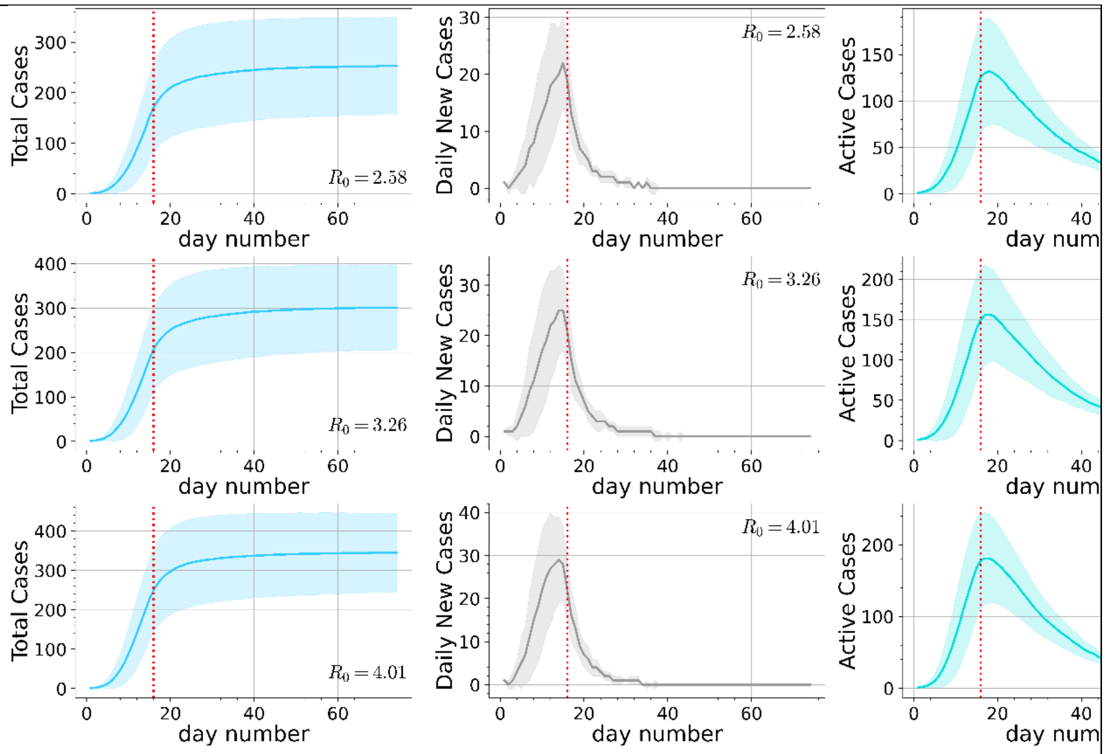


**Figure S 3** Dynamic SEIR simulation results on the synthetic human proximity networks generated with the dynamic-S1 model using the non-normalized number of contacts (i.e. raw number of contacts reported by the study respondents). Top row: total cases, daily new cases and active cases, averaged over 50 dynamic SEIR processes for $R_0$=2.58. Middle and bottom rows show the same results as the top row but for $R_0$=3.26 and $R_0$=4.01, respectively. The shaded area in each plot corresponds to one standard deviation away from the average. The vertical red line marks the beginning of the measures.

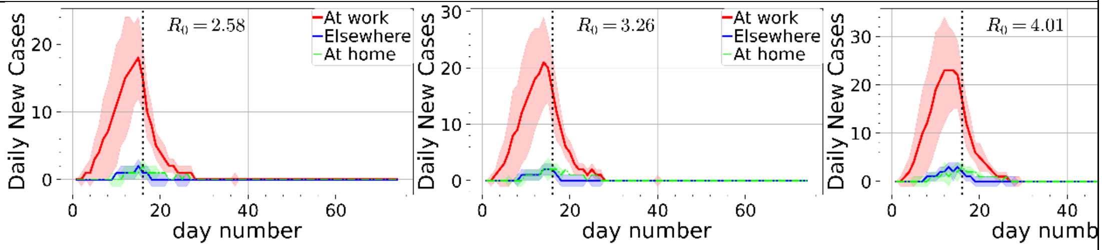


**Figure S 4** Daily new cases originated at work (red lines), elsewhere (blue lines) and at home (dashed green lines), averaged over 50 dynamic SEIR processes on synthetic human proximity networks using the non-normalized number of contacts (i.e. raw number of contacts reported by the study respondents). Cases originated elsewhere and at home are approximately zero after averaging. The shaded areas in each plot correspond to one standard deviation away from the average. The vertical black line marks the beginning of the measures.

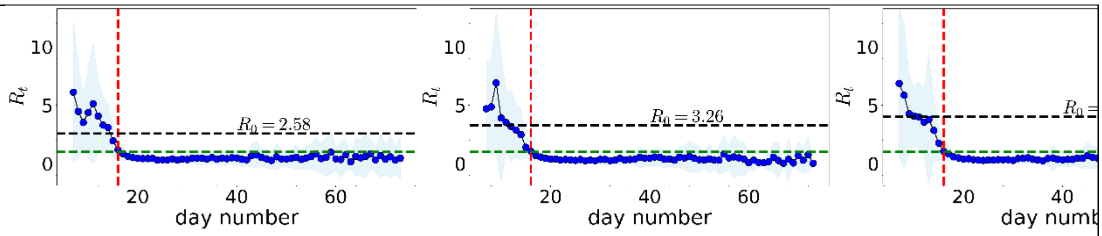


**Figure S 5** Evolution of $R_t$ averaged over 50 dynamic SEIR processes on the synthetic human proximity networks using the non-normalized number of contacts (i.e. raw number of contacts reported by the study respondents). The shaded area in each plot corresponds to one standard deviation away from the average. The green horizontal line marks $R_t = 1$ whereas the horizontal black line marks the corresponding initial $R_0$ value in the simulations. The vertical red line marks the beginning of the measures.

# Methodology and data analysis details

## Data collection

Questions from five validated questionnaires and a diary were used for the design of the selected questions for this proposed study, so that reliability and validity of the questionnaire are established:

1. Diary that documents physical and non-physical contacts, location and duration of each contact (1).
2. European Health Interview Survey for 2014 based on the Cyprus Statistical Services (2)
3. European Urban Health Indicators survey (3)
4. Occupational Survey for Nurses' Health Study in the U.S. (adaptation for questions of washing hands and cleaning house areas) (4)
5. State anxiety inventory STAI form-Y (C.D. Spielberg and validated in GR by Fountoulakis et al.) (5)
6. PSQI sleep questionnaire (Greek version of the Pittsburgh Sleep Quality Index questionnaire) (6)

## The dynamic-$\mathbb{S}^1$ model

The dynamic-$\mathbb{S}^1$ model is based on the $\mathbb{S}^1$ model of traditional complex networks (7,8) and has been recently shown to reproduce many of the observed structural and dynamical properties of human proximity networks (9). The dynamic-$\mathbb{S}^1$ models a sequence of network snapshots, $G_t, t = 1, \ldots, \tau$. Each snapshot is a realization of the $\mathbb{S}^1$ model. Thus, each node in the model has latent variables $\kappa$ and $\theta$, which remain fixed in all snapshots. The latent variable $\kappa$ is the node's hidden degree per time slot, proportional to its expected degree (number of contacts) in each snapshot. Thus, $\kappa$ abstracts the popularity of the node in the population. The latent variable $\theta$ is the angular (similarity) coordinate of the node in a circle of radius $R = N/2\pi$, where $N$ is the total number of nodes. The angular distance $\Delta\theta ij = \pi - |\pi - |\theta i - \theta j||$ between nodes $i, j$ abstracts their similarity. Each snapshot $G_t$ is allowed to have a different average degree $\bar{k}_t, t = 1, \ldots, \tau$. The model also has a network temperature parameter $T$ that takes values in $(0, 1)$. The temperature $T$ plays a central role in network dynamics in the model, dictating the distributions of contact and intercontact durations, the node degrees, among others (9).

To generate a network with $N$ nodes, $\tau$ snapshots, snapshot degrees $\bar{k}_t, t = 1, \ldots, \tau$ and $T \in (0, 1)$ we do the following:

First, for each node $i = 1, 2, \ldots, N$ sample its angular coordinate $\theta_i$ uniformly at random from $[0, 2\pi]$, and its degree variable $\kappa_i$ from a probability density function $\rho(\kappa)$. Then, snapshots are generated according to the following simple rules:Fsha

1. at each time step $t = 1, \ldots, \tau$, snapshot $G_t$ starts with $N$ disconnected nodes.

2. each pair of nodes $i, j$ connects with probability:

$$p(\chi_{ij}) = \frac{1}{1+\chi_{ij}^{1/T}}.$$

In this expression, $\chi_{ij}$ is the effective distance between nodes $i$ and $j$,

$$\chi_{ij} = \frac{R\Delta\theta_{ij}}{\mu\kappa_i\kappa_j},$$

where parameter $\mu$ is derived from the condition that the expected degree in the snapshot is indeed $\bar{k}_t$ yielding

$$\mu = \frac{\bar{k}_t \sin(T\pi)}{2\bar{\kappa}^2 T\pi},$$

where $\bar{\kappa} = \int \kappa\rho(\kappa)d\kappa$.

3. at time $t + 1$, all edges in snapshot $G_t$ are deleted and the process starts over again to generate snapshot $G_{t+1}$.

We note that smaller values of $T$ increase (decrease) the connection probability among nodes at smaller (larger) effective distances.

## Constructing synthetic human proximity networks from the Exposome@home survey

To generate the temporal network for a single setting (workplace, elsewhere or home) before or during the measures with the dynamic-$\mathbb{S}^1$, the procedure is as follows. First, we assume that all considered 578 respondents are present in all settings, therefore we set the number of nodes $N = 578$ in all cases. The number of time slots $\tau$ is set according to the average number of hours spent in the setting in a day reported by the respondents. We assume that the network time-slots have a duration of five minutes, giving $\tau = 84$ (7 hours) at work, $\tau = 12$ (1 hour) elsewhere and $\tau = 144$ (12 hours) at home before the measures, while $\tau$ =24 (2 hours) at work, $\tau = 0$ elsewhere and $\tau = 252$ (21 hours) at home during the measures. The average degree (number of contacts) per time slot is assumed the same in all time slots of the setting $\bar{k}_t = \bar{\kappa}, t = 1, \dots, \tau$. The hidden degrees per time slot $\kappa_i$ of each node $i = 1, \dots, N$, and the temperature $T$ are tuned simultaneously for each setting. The hidden degree per time slot $\kappa_i$ of node i can be estimated as $\kappa_i = k^i_{aggr}/\alpha$, where $\alpha = \tau^T/\Gamma(1 + T)$ and $k^i_{aggr}$ is node $i$'s expected degree aggregated over all time slots, i.e., the node's reported number of contacts in the setting from the Exposome@home survey (9). Using these formulas, we set the hidden degrees $\kappa_i$ of the nodes by experimentally finding the value of $T$ for which the average node degree in the resulting network, aggregated over all time slots, is similar to the average number of contacts reported by the respondents in the corresponding setting in the real data. Before the measures we found that $T = 0.3$ at work and $T = 0.5$ elsewhere and at home. During the measures $T = 0.2$ at work and $T = 0.5$ at home. Finally, the similarity coordinate $\theta_i$ of each node $i$ is sampled uniformly at random from $0$ to $2\pi$ for each setting, and we assume that it remains the same before and during the measures.

## Computation of the serial interval probability $w_s$

To compute the evolution of $R_t$ in each run of the dynamic SEIR process, we use the equation $R_t = I_t/(\sum_{s=1}^{t} I_{t-s} w_s)$, where $I_t$ is the number of new infected cases on day $t$ and $w_s$ is the probability that $s$ days separate the onset of symptoms in an infected individual and the onset of symptoms in its infector—the serial interval— (10). For each infected node $i$ in the simulations, we find the number of

days $s$ that elapsed between the day $i$ transitioned to the infected compartment (onset of symptoms) and the day when the node that infected $i$ transitioned to the infected compartment. Having found all serial intervals $s$ in the simulations we compute the empirical distribution of serial intervals, from which we obtain the probabilities $w_s$.

## References


1. Mossong J, Hens N, Jit M, Beutels P, Auranen K, Mikolajczyk R, et al. Social Contacts and Mixing Patterns Relevant to the Spread of Infectious Diseases. Riley S, editor. PLoS Med. 2008 Mar 25;5(3):e74.

2. Republic of Cyprus, Ministry of Finance. European Health Interview Survey [Internet]. 2014 [cited 2020 Mar 18]. Available from: https://library.cystat.gov.cy/Documents/Questionnaires/EUROPEAN_HEALTH_SURVEY-2014-EL.pdf

3. European Urban Health Indications System Part 2. EURO-URHIS [Internet]. 2012 [cited 2020 Mar 18]. Available from: https://web.archive.org/web/20190308170137/http://results.urhis.eu/Default.aspx?Code=YY&Group=B

4. Nurses' Health Study. Nurses' Health Studies questionnaires [Internet]. 2019 [cited 2020 Mar 18]. Available from: https://nurseshealthstudy.org/participants/questionnaires

5. Fountoulakis KN, Papadopoulou M, Kleanthous S, Papadopoulou A, Bizeli V, Nimatoudis I, et al. Reliability and psychometric properties of the Greek translation of the State-Trait Anxiety Inventory form Y: Preliminary data. Ann Gen Psychiatry. 2006 Dec;5(1):2.

6. Kotronoulas GC, Papadopoulou CN, Papapetrou A, Patiraki E. Psychometric evaluation and feasibility of the Greek Pittsburgh Sleep Quality Index (GR-PSQI) in patients with cancer receiving chemotherapy. Support Care Cancer. 2011 Nov;19(11):1831–40.

7. Serrano MÁ, Krioukov D, Boguñá M. Self-Similarity of Complex Networks and Hidden Metric Spaces. Phys Rev Lett. 2008 Feb 20;100(7):078701.

8. Krioukov D, Papadopoulos F, Kitsak M, Vahdat A, Boguñá M. Hyperbolic geometry of complex networks. Phys Rev E. 2010 Sep 9;82(3):036106.

9. Papadopoulos F, Flores MAR. Latent geometry and dynamics of proximity networks. Phys Rev E. 2019 Nov 26;100(5):052313.

10. Thompson RN, Stockwin JE, van Gaalen RD, Polonsky JA, Kamvar ZN, Demarsh PA, et al. Improved inference of time-varying reproduction numbers during infectious disease outbreaks. Epidemics. 2019 Dec;29:100356.

## Session information and R packages used in the main analysis

```
Session information
R version 4.1.0 (2021-05-18)
Platform: x86_64-w64-mingw32/x64 (64-bit)
Running under: Windows 10 x64 (build 19042)

Matrix products: default

locale:
[1] LC_COLLATE=English_United States.1252
[2] LC_CTYPE=English_United States.1252
[3] LC_MONETARY=English_United States.1252
[4] LC_NUMERIC=C
[5] LC_TIME=English_United States.1252

attached base packages:
[1] grid      stats
[3] graphics  grDevices
[5] utils     datasets
[7] methods   base

other attached packages:
 [1] gdtools_0.2.3
 [2] socialmixr_0.1.8
 [3] wesanderson_0.3.6
 [4] ggraph_2.0.5
 [5] igraph_1.2.6
 [6] corrr_0.4.3
 [7] broom_0.7.8
 [8] glue_1.4.2
 [9] inspectdf_0.0.11
[10] dplyr_1.0.7
[11] purrr_0.3.4
[12] tidyr_1.1.3
[13] tibble_3.1.2
[14] tidyverse_1.3.1
[15] srvyr_1.0.1
[16] survey_4.0
[17] survival_3.2-11
[18] Matrix_1.3-3
[19] rvg_0.2.5
[20] officer_0.3.18
[21] scales_1.1.1
[22] patchwork_1.1.1
[23] corrplot_0.90
[24] circlize_0.4.13
[25] stringr_1.4.0
[26] forcats_0.5.1
[27] janitor_2.1.0
[28] eeptools_1.2.4
[29] ggplot2_3.3.5
```

```
[30] lubridate_1.7.10
[31] sjPlot_2.8.8
[32] psych_2.1.6
[33] readr_1.4.0
[34] stargazer_5.2.2
[35] tableone_0.12.0
[36] knitr_1.33
[37] readxl_1.3.1
```

## References to the R packages used


Alastair Rushworth (2021). inspectdf: Inspection, Comparison and Visualisation of Data Frames. R package version 0.0.11. https://CRAN.R-project.org/package=inspectdf

Csardi G, Nepusz T: The igraph software package for complex network research, InterJournal, Complex Systems 1695. 2006. https://igraph.org

David Gohel (2020). rvg: R Graphics Devices for Vector Graphics Output. R package version 0.2.5. https://CRAN.R-project.org/package=rvg

David Gohel (2021). officer: Manipulation of Microsoft Word and PowerPoint Documents. R package version 0.3.18. https://CRAN.R-project.org/package=officer

David Gohel, Hadley Wickham, Lionel Henry and Jeroen Ooms (2021). gdtools: Utilities for Graphical Rendering. R package version 0.2.3. https://CRAN.R-project.org/package=gdtools

David Robinson, Alex Hayes and Simon Couch (2021). broom: Convert Statistical Objects into Tidy Tibbles. R package version 0.7.8. https://CRAN.R-project.org/package=broom

Douglas Bates and Martin Maechler (2021). Matrix: Sparse and Dense Matrix Classes and Methods. R package version 1.3-3. https://CRAN.R-project.org/package=Matrix

Garrett Grolemund, Hadley Wickham (2011). Dates and Times Made Easy with lubridate. Journal of Statistical Software, 40(3), 1-25. URL https://www.jstatsoft.org/v40/i03/.

Greg Freedman Ellis and Ben Schneider (2021). srvyr: 'dplyr'-Like Syntax for Summary Statistics of Survey Data. R package version 1.0.1. https://CRAN.R-project.org/package=srvyr

Gu, Z. (2014) circlize implements and enhances circular visualization in R. Bioinformatics.

H. Wickham. ggplot2: Elegant Graphics for Data Analysis. Springer-Verlag New York, 2016.

Hadley Wickham (2019). stringr: Simple, Consistent Wrappers for Common String Operations. R package version 1.4.0. https://CRAN.R-project.org/package=stringr

Hadley Wickham (2021). forcats: Tools for Working with Categorical Variables (Factors). R package version 0.5.1. https://CRAN.R-project.org/package=forcats

Hadley Wickham (2021). tidyr: Tidy Messy Data. R package version 1.1.3. https://CRAN.R-project.org/package=tidyr

Hadley Wickham and Dana Seidel (2020). scales: Scale Functions for Visualization. R package version 1.1.1. https://CRAN.R-project.org/package=scales

Hadley Wickham and Jennifer Bryan (2019). readxl: Read Excel Files. R package version 1.3.1. https://CRAN.R-project.org/package=readxl

Hadley Wickham and Jim Hester (2020). readr: Read Rectangular Text Data. R package version 1.4.0. https://CRAN.R-project.org/package=readr

Hadley Wickham, Romain François, Lionel Henry and Kirill Müller (2021). dplyr: A Grammar of Data Manipulation. R package version 1.0.7. https://CRAN.R-project.org/package=dplyr

Hlavac, Marek (2018). stargazer: Well-Formatted Regression and Summary Statistics Tables. R package version 5.2.1. https://CRAN.R-project.org/package=stargazer

Jared E. Knowles (2020). eeptools: Convenience Functions for Education Data. R package version 1.2.4. https://CRAN.R-project.org/package=eeptools

Jim Hester (2020). glue: Interpreted String Literals. R package version 1.4.2. https://CRAN.R-project.org/package=glue

Karthik Ram and Hadley Wickham (2018). wesanderson: A Wes Anderson Palette Generator. R package version 0.3.6. https://CRAN.R-project.org/package=wesanderson

Kazuki Yoshida and Alexander Bartel (2020). tableone: Create 'Table 1' to Describe Baseline Characteristics with or without Propensity Score Weights. R package version 0.12.0. https://CRAN.R-project.org/package=tableone

Kirill Müller and Hadley Wickham (2021). tibble: Simple Data Frames. R package version 3.1.2. https://CRAN.R-project.org/package=tibble

Lionel Henry and Hadley Wickham (2020). purrr: Functional Programming Tools. R package version 0.3.4. https://CRAN.R-project.org/package=purrr

Lüdecke D (2021)._sjPlot: DataVisualization forStatistics in SocialScience_. R packageversion 2.8.8, <URL:https://CRAN.R-project.org/package=sjPlot>.

Max Kuhn, Simon Jackson and Jorge Cimentada (2020). corrr: Correlations in R. R package version 0.4.3. https://CRAN.R-project.org/package=corrr

R Core Team (2021). R: A language and environment for statistical computing. R Foundation for Statistical Computing, Vienna, Austria. URL https://www.R-project.org/.

Revelle, W. (2021) psych: Procedures for Personality and Psychological Research, Northwestern University, Evanston, Illinois, USA, https://CRAN.R-project.org/package=psych Version = 2.1.6,.

Sam Firke (2021). janitor: Simple Tools for Examining and Cleaning Dirty Data. R package version 2.1.0. https://CRAN.R-project.org/package=janitor

Sebastian Funk (2020). socialmixr: Social Mixing Matrices for Infectious Disease Modelling. R package version 0.1.8. https://CRAN.R-project.org/package=socialmixr

T. Lumley (2020) "survey: analysis of complex survey samples". R package version 4.0.

Taiyun Wei and Viliam Simko (2021). R package ‘corrplot’: Visualization of a Correlation Matrix (Version 0.90). Available from https://github.com/taiyun/corrplot

Therneau T (2021)._A Package forSurvival Analysis inR_. R packageversion 3.2-11,<URL:https://CRAN.R-project.org/package=survival>.

Thomas Lin Pedersen (2020). patchwork: The Composer of Plots. R package version 1.1.1. https://CRAN.R-project.org/package=patchwork

Thomas Lin Pedersen (2021). ggraph: An Implementation of Grammar of Graphics for Graphs and Networks. R package version 2.0.5. https://CRAN.R-project.org/package=ggraph

Wickham et al., (2019). Welcome to the tidyverse. Journal of Open Source Software, 4(43), 1686, https://doi.org/10.21105/joss.01686

Yihui Xie (2021). knitr: A General-Purpose Package for Dynamic Report Generation in R. R package version 1.33.

**Checklist for Reporting Results of Internet E-Surveys (CHERRIES) [Source: https://www.equator-network.org/reporting-guidelines/improving-the-quality-of-web-surveys-the-checklist-for-reporting-results-of-internet-e-surveys-cherries/, Citation: Eysenbach G. Improving the quality of Web surveys: the Checklist for Reporting Results of Internet E-Surveys (CHERRIES). J Med Internet Res. 2004;6:e34.]**

| *Checklist Item* | *Explanation* | *Description* |
|---|---|---|
| Describe survey design | Describe target population, sample frame. Is the sample a convenience sample? (In "open" surveys this is most likely.) | The Exposome@home\|COVID-19 was an online survey. The survey questionnaire was administered during the time physical distancing measures and stay-at-home orders were implemented during the 1st wave of the COVID-19 pandemic (30 March-4 May 2020). Respondents were informed about the study through university mailing lists and through posts and advertisements on social media and they were also invited to forward information about the study and the questionnaire to their contacts. |
| IRB approval | Mention whether the study has been approved by an IRB. | The study was approved by the Cyprus National Bioethics Committee (ΕΕΒΚ/ΕΠ/2020.01.52). |
| Informed consent | Describe the informed consent process. Where were the participants told the length of time of the survey, which data were stored and where and for how long, who the investigator was, and the purpose of the study? | Respondents were informed about length of time of the survey, the duration of data storage, the responsible investigator, and the purpose of the study on the cover page of the questionnaire. Also, parents were informed that the data collection was anonymous and that, if they wished to, they would withdraw from the study at any time during survey completion step. |

| | | |
|---|---|---|
| Data protection | If any personal information was collected or stored, describe what mechanisms were used to protect unauthorized access. | Respondents were informed that all collected data will be used for statistical analysis along without showing any personal data. Only the research team has access to the data using a password-based database at the Cyprus International Institute for Environmental and Public Health. |
| Development and testing | State how the survey was developed, including whether the usability and technical functionality of the electronic questionnaire had been tested before fielding the questionnaire. | Data was collected and managed using KOBO Toolbox |
| Open survey versus closed survey | An "open survey" is a survey open for each visitor of a site, while a closed survey is only open to a sample which the investigator knows (password-protected survey). | Open survey (no password needed) |
| Contact mode | Indicate whether or not the initial contact with the potential participants was made on the Internet. (Investigators may also send out questionnaires by mail and allow for Web-based data entry.) | Respondents were informed about the study through university mailing lists and through posts and advertisements on social media and they were also invited to forward information about the study and the questionnaire to their contacts. |
| Advertising the survey | How/where was the survey announced or advertised? Some examples are offline media (newspapers), or online (mailing lists – If yes, which ones?) or banner ads (Where were these banner ads posted and what did they look like?). It is important to know the wording of the announcement as it | The survey was forwarded using email (university mailing lists) and posts in social media. The survey was advertised in Facebook for 5 days (15-20/04/2020). |

| | | |
|---|---|---|
| | will heavily influence who chooses to participate. Ideally the survey announcement should be published as an appendix. | |
| Web/E-mail | State the type of e-survey (eg, one posted on a Web site, or one sent out through e-mail). If it is an e-mail survey, were the responses entered manually into a database, or was there an automatic method for capturing responses? | Data was collected and managed using KOBO Toolbox |
| Context | Describe the Web site (for mailing list/newsgroup) in which the survey was posted. What is the Web site about, who is visiting it, what are visitors normally looking for? Discuss to what degree the content of the Web site could pre-select the sample or influence the results. For example, a survey about vaccination on a anti-immunization Web site will have different results from a Web survey conducted on a government Web site | We posted the survey in Facebook and emailed contacts from the Cyprus University of Technology mailing list |
| Mandatory/voluntary | Was it a mandatory survey to be filled in by every visitor who wanted to enter the Web site, or was it a voluntary survey? | Voluntary survey |
| Incentives | Were any incentives offered (eg, monetary, prizes, or non-monetary incentives such as an offer to provide the survey results)? | No incentive was offered |
| Time/Date | In what timeframe were the data collected? | 30/03/2020-04/05/2020 |

| | | |
|---|---|---|
| Randomization of items or questionnaires | To prevent biases items can be randomized or alternated. | No randomization or alternation of items |
| Adaptive questioning | Use adaptive questioning (certain items, or only conditionally displayed based on responses to other items) to reduce number and complexity of the questions. | Adaptive questioning was used- some questions were conditionally displayed based on responses to previous questions |
| Number of Items | What was the number of questionnaire items per page? The number of items is an important factor for the completion rate. | Different number of questions based on questionnaire section |
| Number of screens (pages) | Over how many pages was the questionnaire distributed? The number of items is an important factor for the completion rate. | 12 pages, including the cover page |
| Completeness check | It is technically possible to do consistency or completeness checks before the questionnaire is submitted. Was this done, and if “yes”, how (usually JAVAScript)? An alternative is to check for completeness after the questionnaire has been submitted (and highlight mandatory items). If this has been done, it should be reported. All items should provide a non-response option such as “not applicable” or “rather not say”, and selection of one response option should be enforced. | Most questions were mandatory and the options of Don’t know/Don’t want to answer were included as options. |

| | | |
|---|---|---|
| Review step | State whether respondents were able to review and change their answers (eg, through a Back button or a Review step which displays a summary of the responses and asks the respondents if they are correct). | Respondents were able to change their answers prior to submitting by pressing the back button |
| Unique site visitor | If you provide view rates or participation rates, you need to define how you determined a unique visitor. There are different techniques available, based on IP addresses or cookies or both. | Not applicable |
| View rate (Ratio of unique survey visitors/unique site visitors) | Requires counting unique visitors to the first page of the survey, divided by the number of unique site visitors (not page views!). It is not unusual to have view rates of less than 0.1 % if the survey is voluntary. | Not applicable |
| Participation rate (Ratio of unique visitors who agreed to participate/unique first survey page visitors) | Count the unique number of people who filled in the first survey page (or agreed to participate, for example by checking a checkbox), divided by visitors who visit the first page of the survey (or the informed consents page, if present). This can also be called "recruitment" rate. | Not applicable |
| Completion rate (Ratio of users who finished the survey/users who | The number of people submitting the last questionnaire page, divided by the number of people who agreed to participate (or submitted the first survey page). This is only relevant if there is a separate "informed consent" page or if | Not applicable |

| | | |
|---|---|---|
| agreed to participate) | the survey goes over several pages. This is a measure for attrition. Note that “completion” can involve leaving questionnaire items blank. This is not a measure for how completely questionnaires were filled in. (If you need a measure for this, use the word “completeness rate”.) | |
| Cookies used | Indicate whether cookies were used to assign a unique user identifier to each client computer. If so, mention the page on which the cookie was set and read, and how long the cookie was valid. Were duplicate entries avoided by preventing users access to the survey twice; or were duplicate database entries having the same user ID eliminated before analysis? In the latter case, which entries were kept for analysis (eg, the first entry or the most recent)? | Not applicable |
| IP check | Indicate whether the IP address of the client computer was used to identify potential duplicate entries from the same user. If so, mention the period of time for which no two entries from the same IP address were allowed (eg, 24 hours). Were duplicate entries avoided by preventing users with the same IP address access to the survey twice; or were duplicate database entries having the same IP address within a given period of time eliminated before analysis? If | Not applicable |

| | | |
|---|---|---|
| | the latter, which entries were kept for analysis (eg, the first entry or the most recent)? | |
| Log file analysis | Indicate whether other techniques to analyze the log file for identification of multiple entries were used. If so, please describe. | Not applicable |
| Registration | In “closed” (non-open) surveys, users need to login first and it is easier to prevent duplicate entries from the same user. Describe how this was done. For example, was the survey never displayed a second time once the user had filled it in, or was the username stored together with the survey results and later eliminated? If the latter, which entries were kept for analysis (eg, the first entry or the most recent)? | Not applicable |
| Handling of incomplete questionnaires | Were only completed questionnaires analyzed? Were questionnaires which terminated early (where, for example, users did not go through all questionnaire pages) also analyzed? | Only completed questionnaires were analyzed |
| Questionnaires submitted with an atypical timestamp | Some investigators may measure the time people needed to fill in a questionnaire and exclude questionnaires that were submitted too soon. Specify the timeframe that was used as a cut-off point, and describe how this point was determined. | Not applicable |

| | | |
|---|---|---|
| Statistical correction | Indicate whether any methods such as weighting of items or propensity scores have been used to adjust for the non-representative sample; if so, please describe the methods. | To account for differences between the study population and the Cypriot population distribution by age, sex, and geographical district, as well as to allow for extrapolation of the study estimates to the Cypriot population, we weighted the survey population using the raking method. To calculate the weights, we used the most recent (2019) population age and sex estimates by each geographical district available by the Statistical Service of Cyprus. |
